# Supplementary material for: Genetic Deconvolution of Embryonic and Maternal Cell‐Free DNA in Spent Culture Medium of Human Preimplantation Embryo Through Deep Learning
Source: Adv Sci (Weinh). 2025 Jun 29;12(34):e12660. doi: 10.1002/advs.202412660 (PMC12442666; doi:10.1002/advs.202412660)
Supplement: Supplementary file 1 — Supporting Information [file ADVS-12-e12660-s002.pdf]

ADVANCED SCIENCE
Open Access

Supporting Information

for *Adv. Sci.*, DOI 10.1002/advs.202412660

Genetic Deconvolution of Embryonic and Maternal Cell-Free DNA in Spent Culture Medium of Human Preimplantation Embryo Through Deep Learning

Zhenyi Zhang, Jie Qiao, Yidong Chen and Peijie Zhou**

Supplementary Information

Contents

A Supplementary Figures 3

A.1 Supplementary Figure S1: Evaluation of DECENT on Validation Dataset . . . 3

A.2 Supplementary Figure S2: Evaluation of Embryonic CNV Reconstruction in Simulated SECM Samples with Contamination Levels Exceeding 75% . . . 4

A.3 Supplementary Figure S3: Influence of Reads Count on CNV Analysis Results . . . 5

A.4 Supplementary Figure S4: Filter Analysis of DECENT (I) . . . 6

A.5 Supplementary Figure S5: Filter Analysis of DECENT (II) . . . 7

A.6 Supplementary Figure S6: Filter Analysis of DECENT (III) . . . 8

A.7 Supplementary Figure S7: Attribution Analysis of DECENT on Embryonic Reads . . . 9

A.8 Supplementary Figure S8: Attribution Analysis of DECENT on Cumulus-Liked Reads . . . 10

A.9 Supplementary Figure S9: Distribution of high-scoring maternal reads and low-scoring embryonic reads in PCA space across different neural network layers. . . . 11

A.10 Supplementary Figure S10: Attention Weight Distribution of DECENT on Embryonic Reads . . . 12

A.11 Supplementary Figure S11: Attention Weight Distribution of DECENT on Cumulus-Like Reads . . . 13

A.12 Supplementary Figure S12: Effect of DMR Enrichment on Model Classification Performance. . . . 14

A.13 Supplementary Figure S13: Ablation studies for the CNN, LSTM, and attention components. . . . 14

A.14 Supplementary Figure S14: Proportion of DMR Coverage Across Chromosomes. . . . 15

A.15 Supplementary Figure S15: Analysis Results of DECENT on Moderate Contaminated SECM samples (I) . . . 16

A.16 Supplementary Figure S16: Analysis Results of DECENT on Moderate Contaminated SECM samples (II) . . . 17

A.17 Supplementary Figure S17: Analysis Results of DECENT on Moderate Contaminated SECM samples (III) . . . 18

A.18 Supplementary Figure S18: Analysis Results of DECENT on Moderate Contaminated SECM samples (IV) . . . 19

A.19 Supplementary Figure S19: Analysis Results of DECENT on Moderate Contaminated SECM samples (V) . . . 20

A.20 Supplementary Figure S20: Analysis Results of DECENT on Moderate Contaminated SECM samples (VI) . . . 21

A.21 Supplementary Figure S21: Analysis Results of DECENT on Moderate Contaminated SECM samples (VII) . . . 22

A.22 Supplementary Figure S22: Analysis Results of DECENT on Moderate Contaminated SECM samples (VIII) . . . 23

A.23 Supplementary Figure S23: Analysis Results of DECENT on Moderate Contaminated SECM samples (IX) . . . 24

A.24 Supplementary Figure S24: Analysis Results of DECENT on Moderate Contaminated SECM samples (X) . . . 25

A.25 Supplementary Figure S25: Analysis Results of DECENT on Severe Contaminated SECM samples (I) . . . 26

A.26 Supplementary Figure S26: Analysis Results of DECENT on Severe Contaminated SECM samples (II) . . . 27

A.27 Supplementary Figure S27: Analysis Results of DECENT on Severe Contaminated SECM samples (III) . . . 28

A.28 Supplementary Figure S28: Analysis Results of DECENT on Severe Contaminated SECM samples (IV) . . . 29

A.29 Supplementary Figure S29: Analysis Results of DECENT on Severe Contaminated SECM samples (V) . . . 30

A.30 Supplementary Figure S30: Analysis Results of DECENT on Severe Contaminated SECM samples (VI) . . . 31

A.31 Supplementary Figure S31: Analysis Results of DECENT on Severe Contaminated SECM samples (VII) . . . 32

A.32 Supplementary Figure S32: Analysis Results of DECENT on Severe Contaminated SECM samples (VIII) . . . 33

A.33 Supplementary Figure S33: Analysis Results of DECENT on Severe Contaminated SECM samples (IX) . . . 34

A.34 Supplementary Figure S34: Analysis Results of DECENT on Severe Contaminated SECM samples (X) . . . 35

A.35 Supplementary Figure S35: Enrichment analysis using ChIP-seq data from ICM cells and MII oocytes. . . . 36

A.36 Supplementary Figure S36: Differential expression between ICM and cumulus cells. . . . 37

A.37 Supplementary Figure S37: Estimation of maternal contamination proportion. . . . 38

B Supplementary Tables 39

B.1 Supplementary Table 1: DECNET's Architecture and Hyperparameters . . . 39

B.2 Supplementary Table 2: Setting of Hyperparameters Used in the Training Process . . . 39

B.3 Supplementary Table 3: Detailed Read Counts of Mixture Proportions Simulation . . . 40

B.4 Supplementary Table 4: Detailed Profiles of Filtered Moderate SECM Samples . . . 41

B.5 Supplementary Table 5: Detailed Profiles of Filtered Severe SECM Samples . . . 42

B.6 Supplementary Table 6: Ratio of retained reads at zero contamination level using a threshold of 0.15. . . . 43

B.7 Supplementary Table 7: Impact of DMR-Based Preselection on Read Retention in Samples with Low Contamination. 44

A Supplementary Figures

A.1 Supplementary Figure S1: Evaluation of DECENT on Validation Dataset

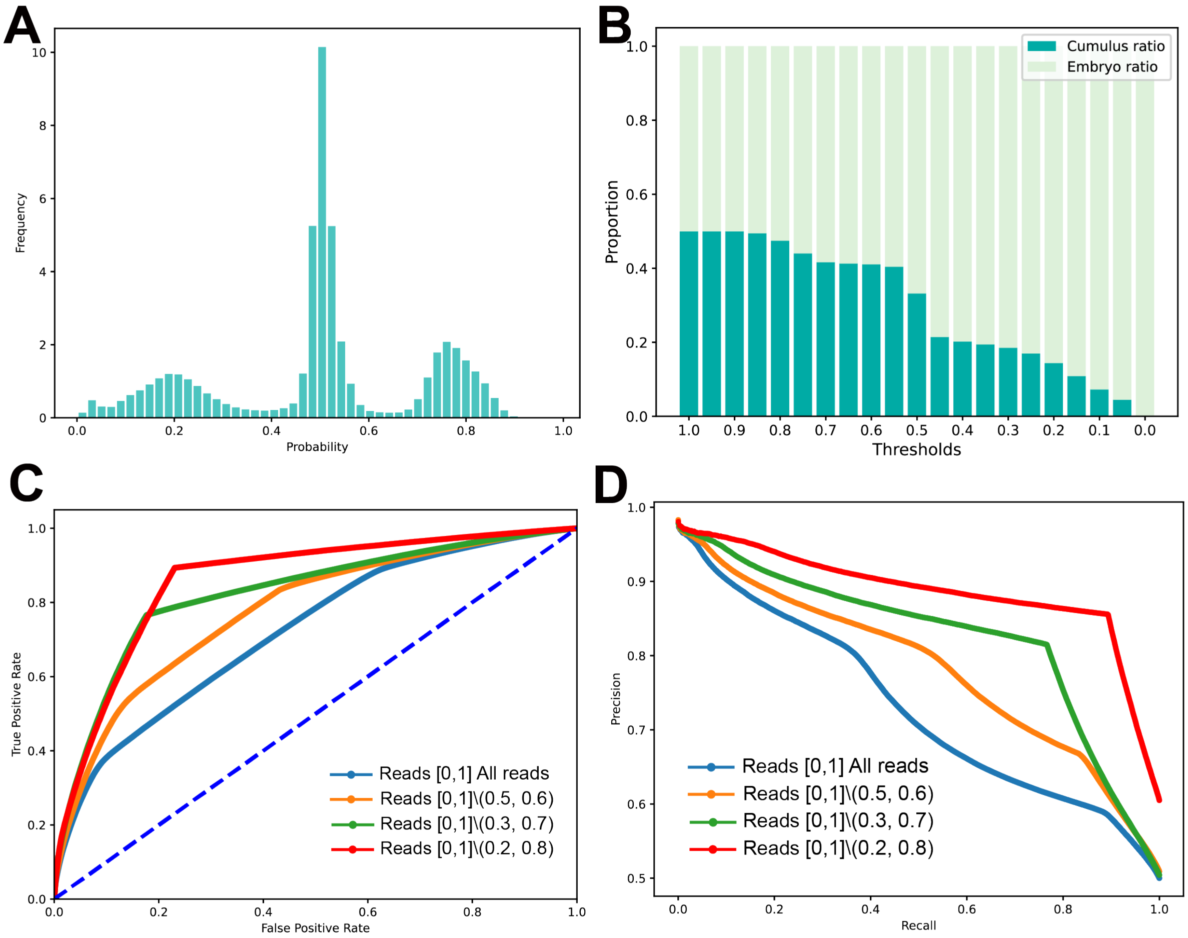

Figure S1. Evaluation of DECENT on the validation dataset. **(A)** Distribution of scores generated by the neural network model on the validation dataset, showing three main modes concentrated around 0.2, 0.5, and 0.8, respectively. **(B)** Variation in identified cumulus contamination proportion with different threshold values on the training dataset. A decrease in the proportion of cumulus contamination is observed as the threshold decreases. **(C)** Receiver operating characteristic (ROC) curves comparing the performance of the deep learning model under different threshold ranges for read exclusion. **(D)** Precision-recall (P-R) curves demonstrating the trade-off between precision and recall for the deep learning model under different threshold ranges for read exclusion.

A.2 Supplementary Figure S2: Evaluation of Embryonic CNV Reconstruction in Simulated SECM Samples with Contamination Levels Exceeding 75%

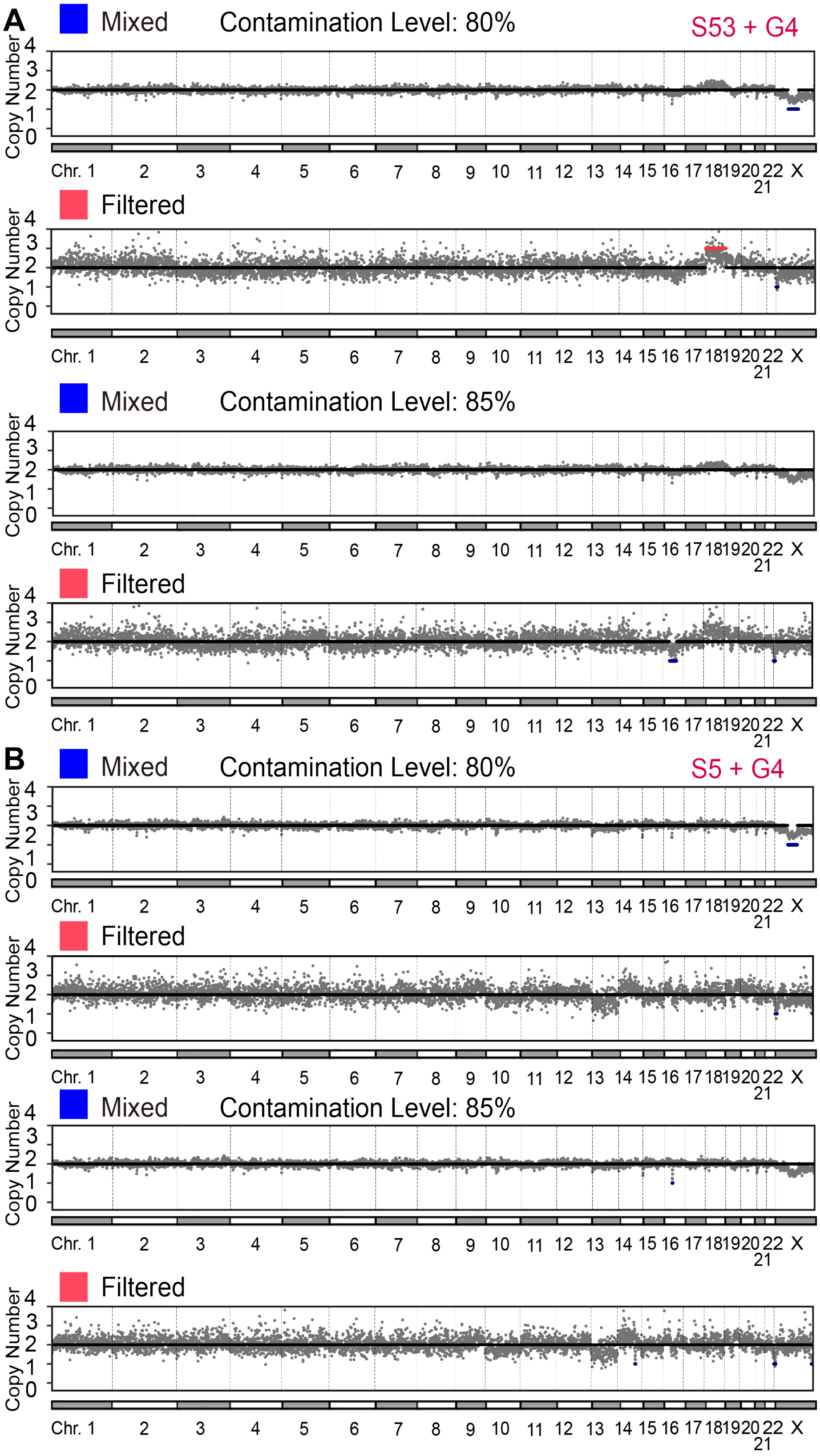

Figure S2. Evaluation of embryonic CNV reconstruction in simulated SECM samples with contamination levels exceeding 75%. **(A)** S53 and G4, **(B)** S5 and G4.

A.3 Supplementary Figure S3: Influence of Reads Count on CNV Analysis Results

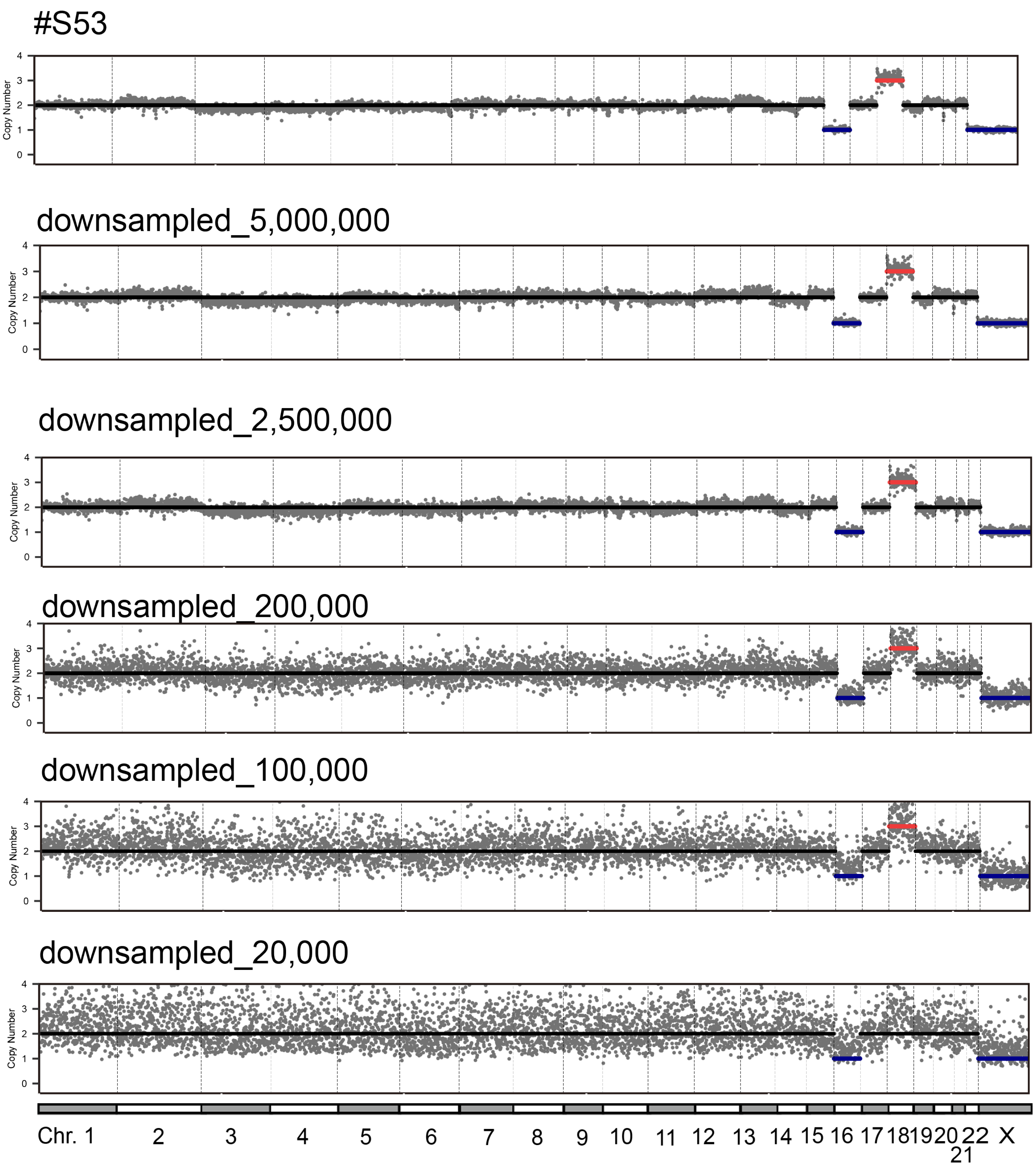

Figure S3. Influence of reads count on CNV analysis results. We selected sample S53 which CNV have -16, +18 profile. Then the reads were downsampled to certain counts respectively, to investigate the effect of read count on CNV analysis results.

A.4 Supplementary Figure S4: Filter Analysis of DECENT (I)

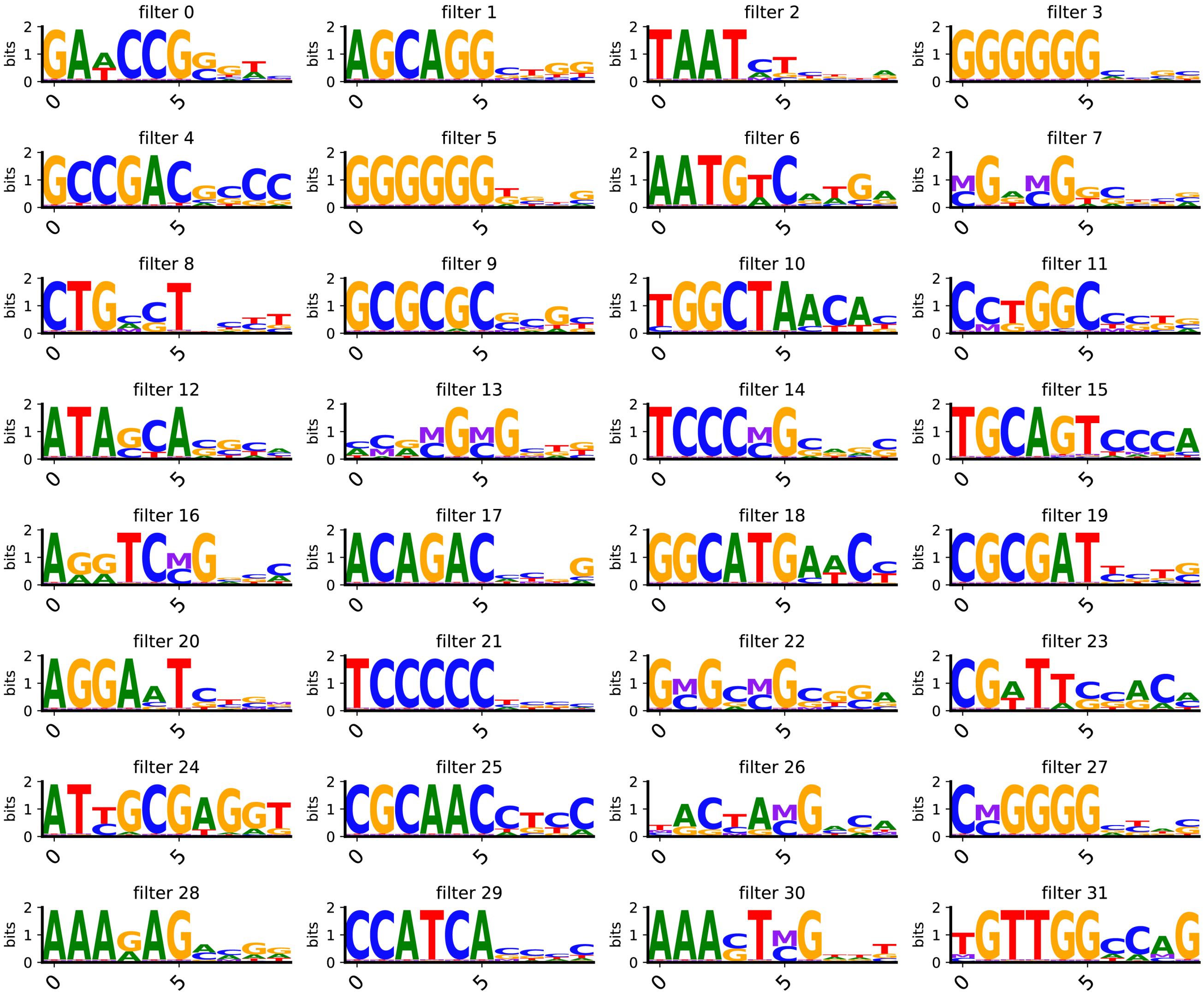

Figure S4. Filter analysis of DECENT (I). Visualization of motif features captured by the first convolutional layer kernels.

A.5 Supplementary Figure S5: Filter Analysis of DECENT (II)

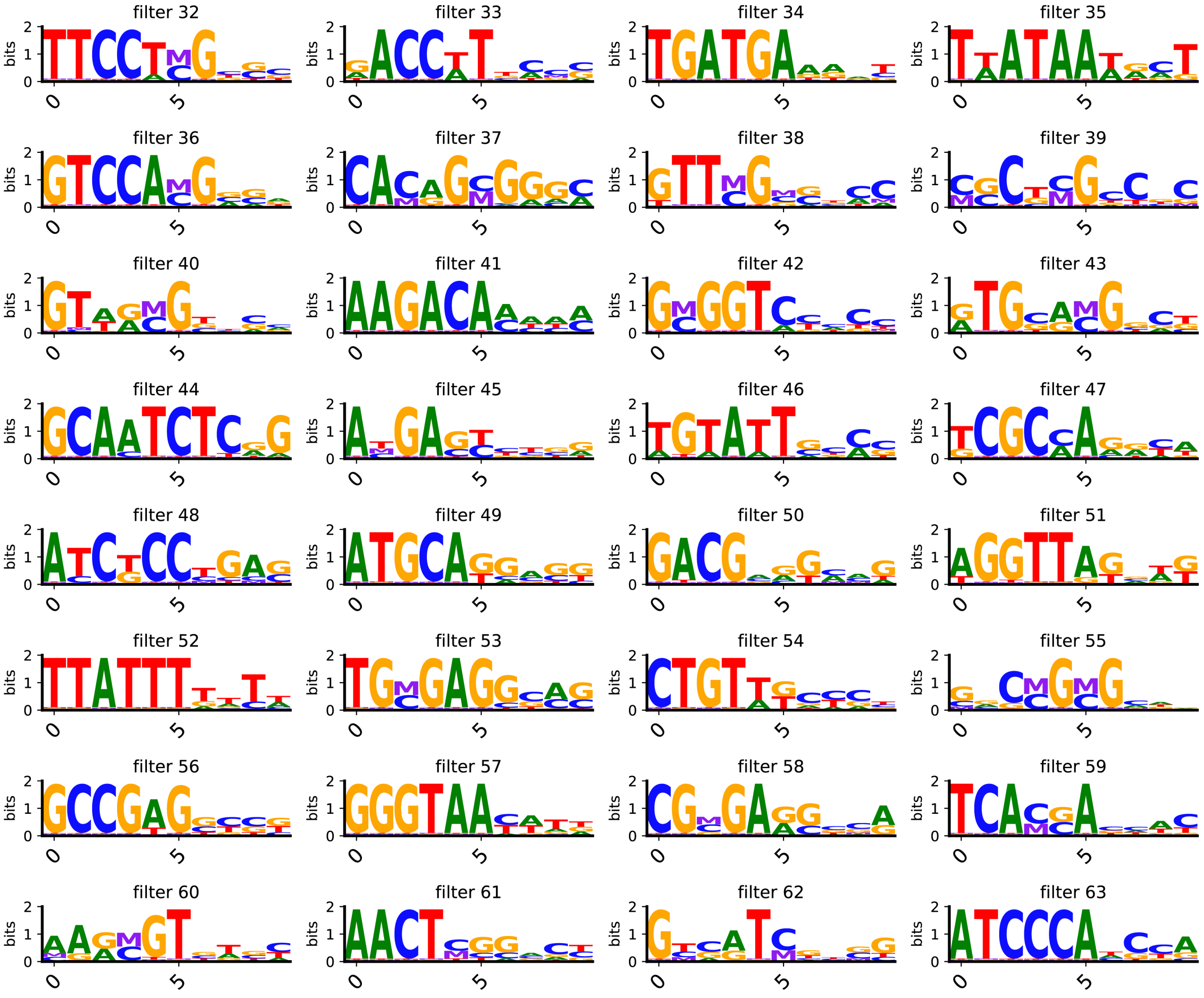

Figure S5. Filter analysis of DECENT (II). Visualization of motif features captured by the first convolutional layer kernels.

A.6 Supplementary Figure S6: Filter Analysis of DECENT (III)

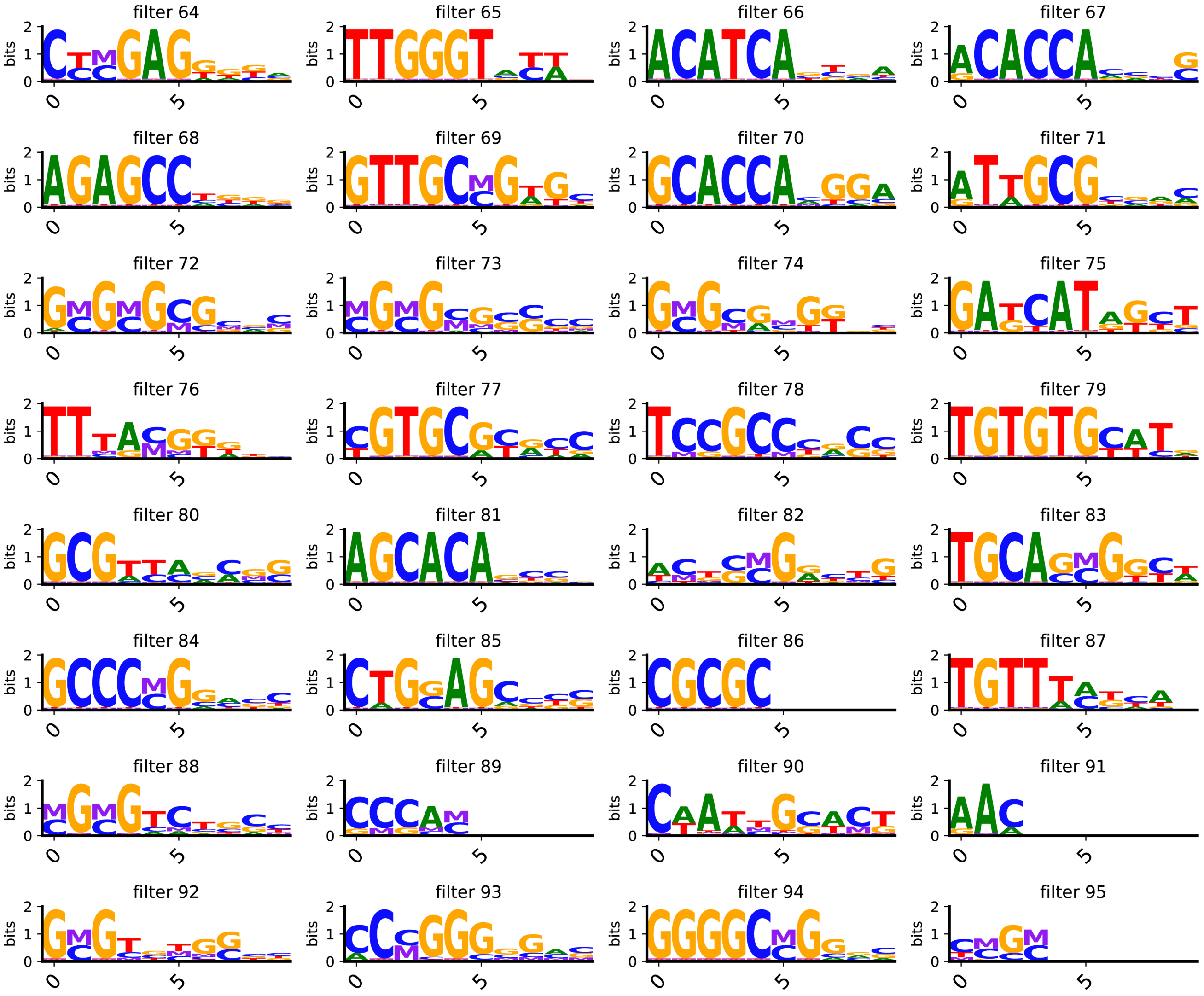

Figure S6. Filter analysis of DECENT (III). Visualization of motif features captured by the first convolutional layer kernels.

A.7 Supplementary Figure S7: Attribution Analysis of DECENT on Embryonic Reads

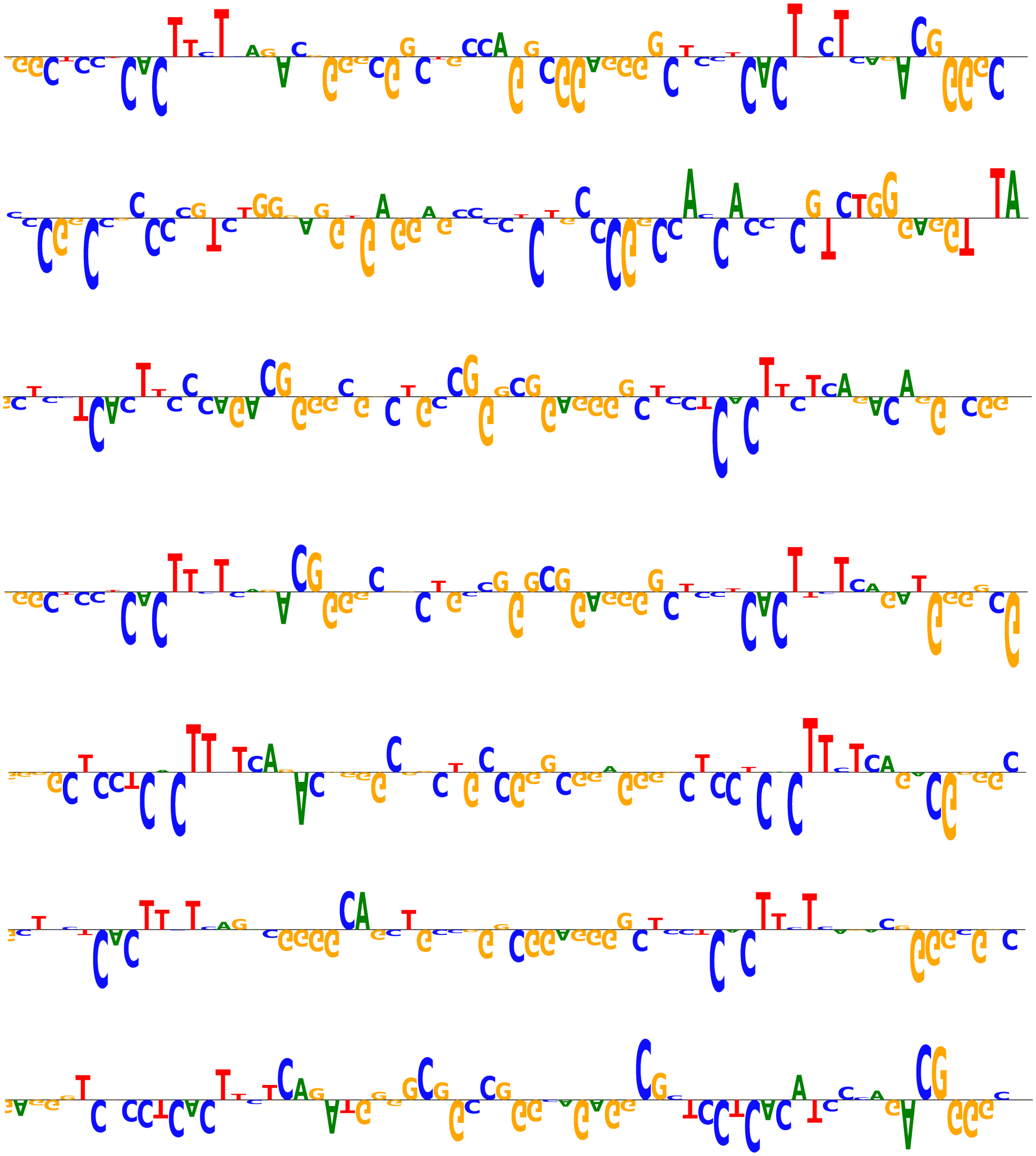

Figure S7. Attribution analysis of DECENT on reads classified as more embryonic-like by the neural network.

A.8 Supplementary Figure S8: Attribution Analysis of DECENT on Cumulus-Liked Reads

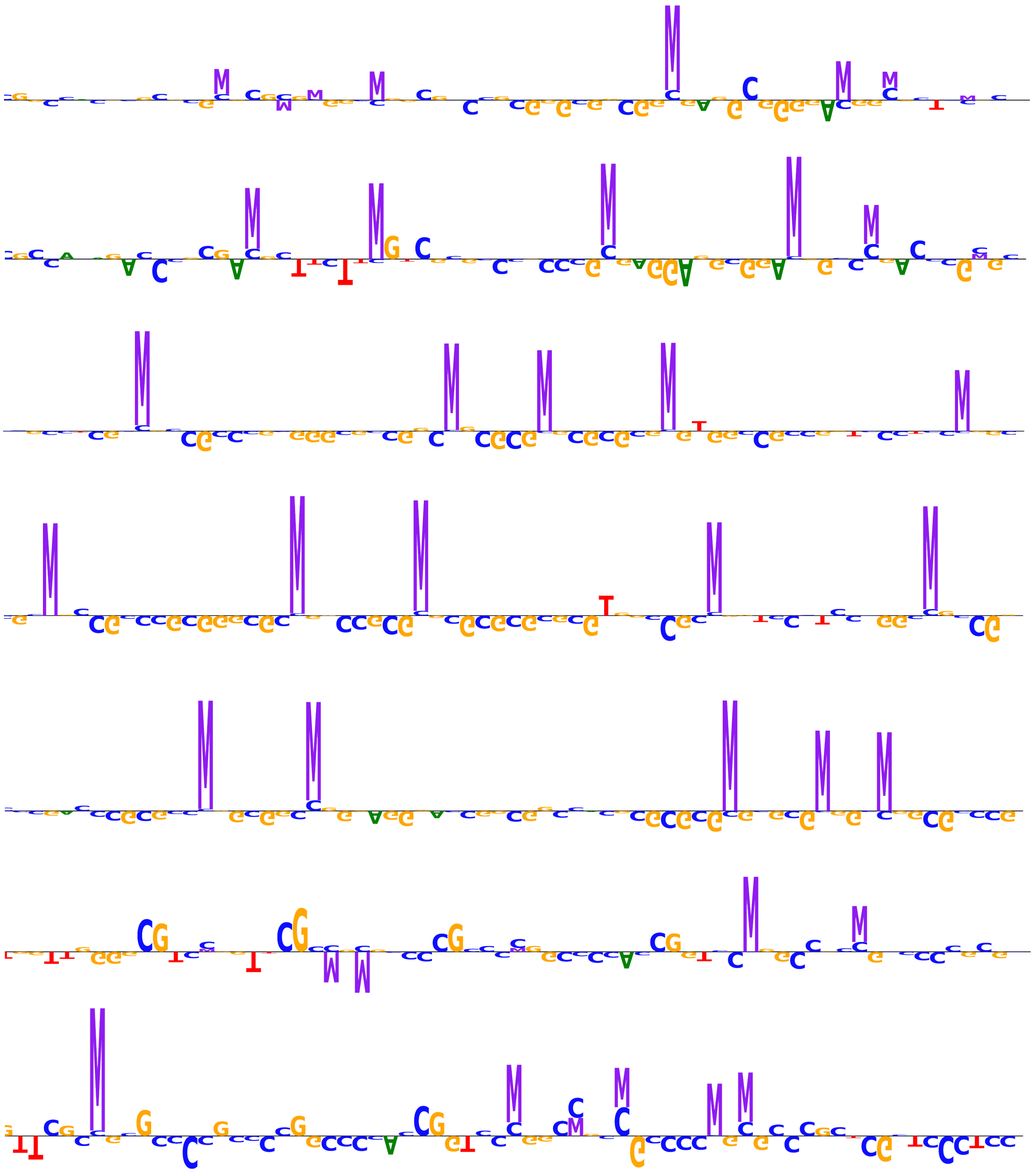

Figure S8. Attribution analysis of DECENT on reads classified as more maternal-like by the neural network.

A.9 Supplementary Figure S9: Distribution of high-scoring maternal reads and low-scoring embryonic reads in PCA space across different neural network layers.

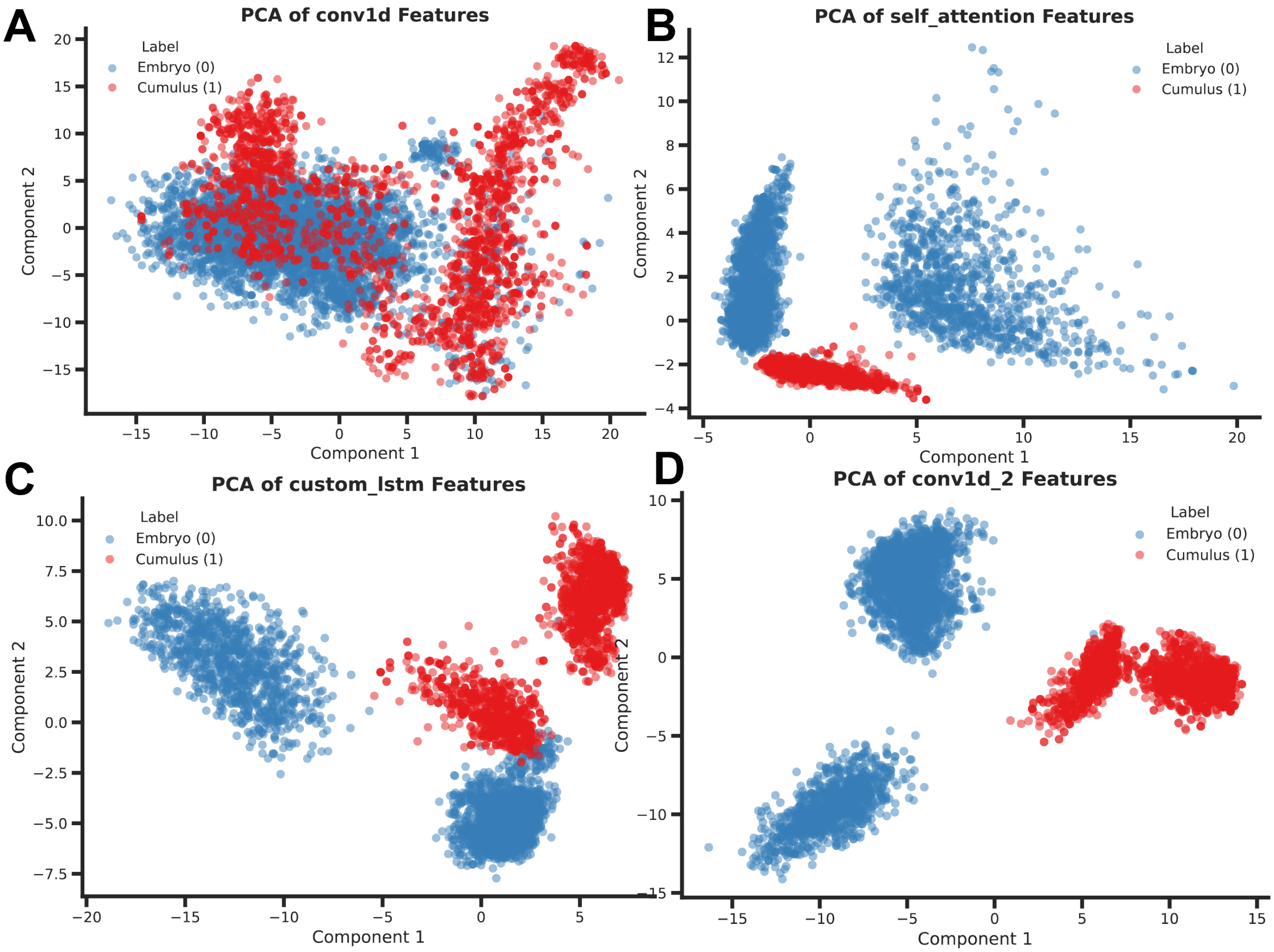

Figure S9. Distribution of high-scoring maternal reads and low-scoring embryonic reads in the principal component space across different neural network layers. (A) The first convolution layer. (B) The attention layer. (C) The LSTM layer. (D) The second convolution layer.

A.10 Supplementary Figure S10: Attention Weight Distribution of DECENT on Embryonic Reads

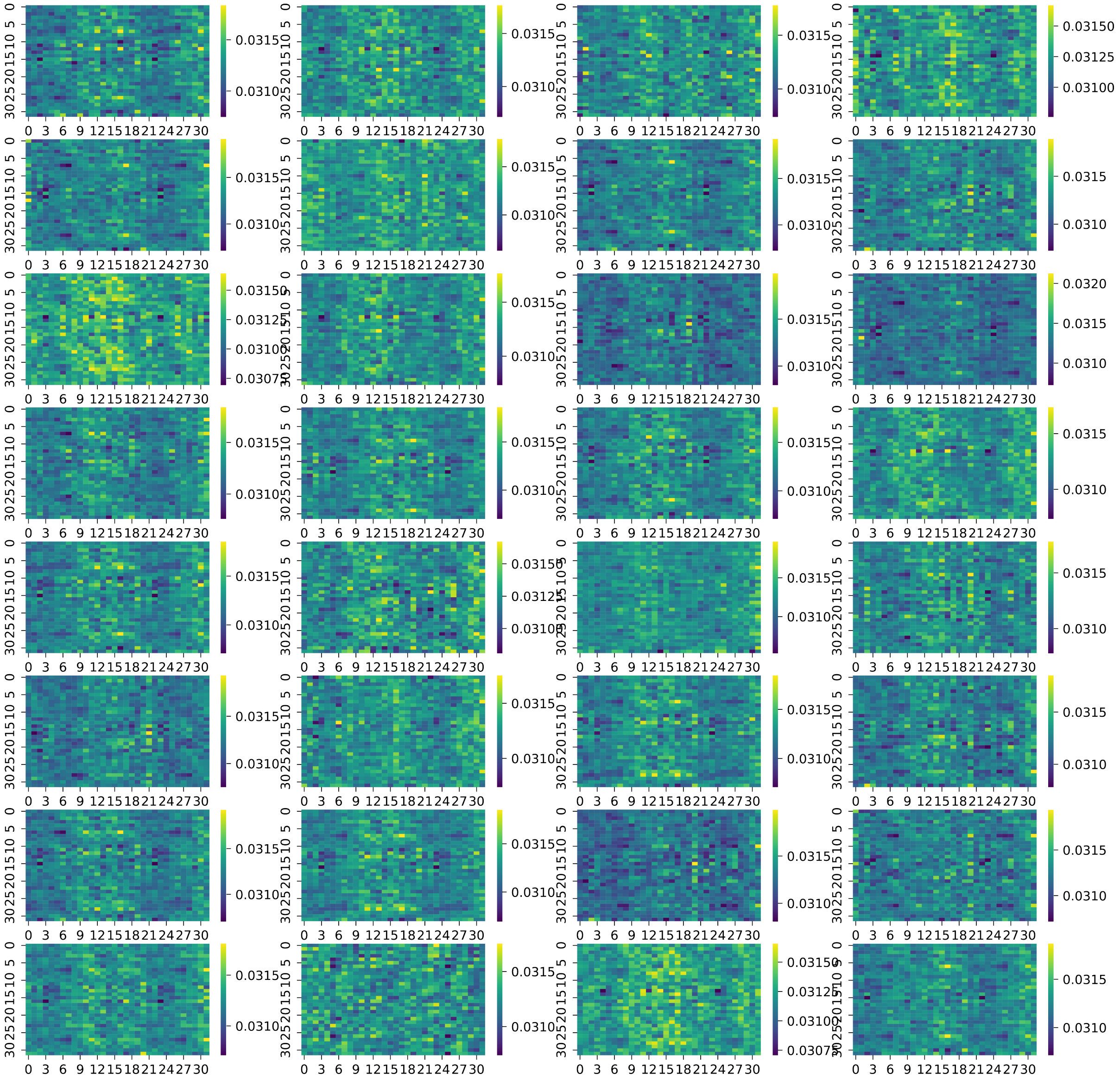

Figure S10. Attention weight distribution of DECENT on embryonic reads. Visualization of average head attention weight matrices captured by the multi-head attention module.

A.11 Supplementary Figure S11: Attention Weight Distribution of DECENT on Cumulus-Like Reads

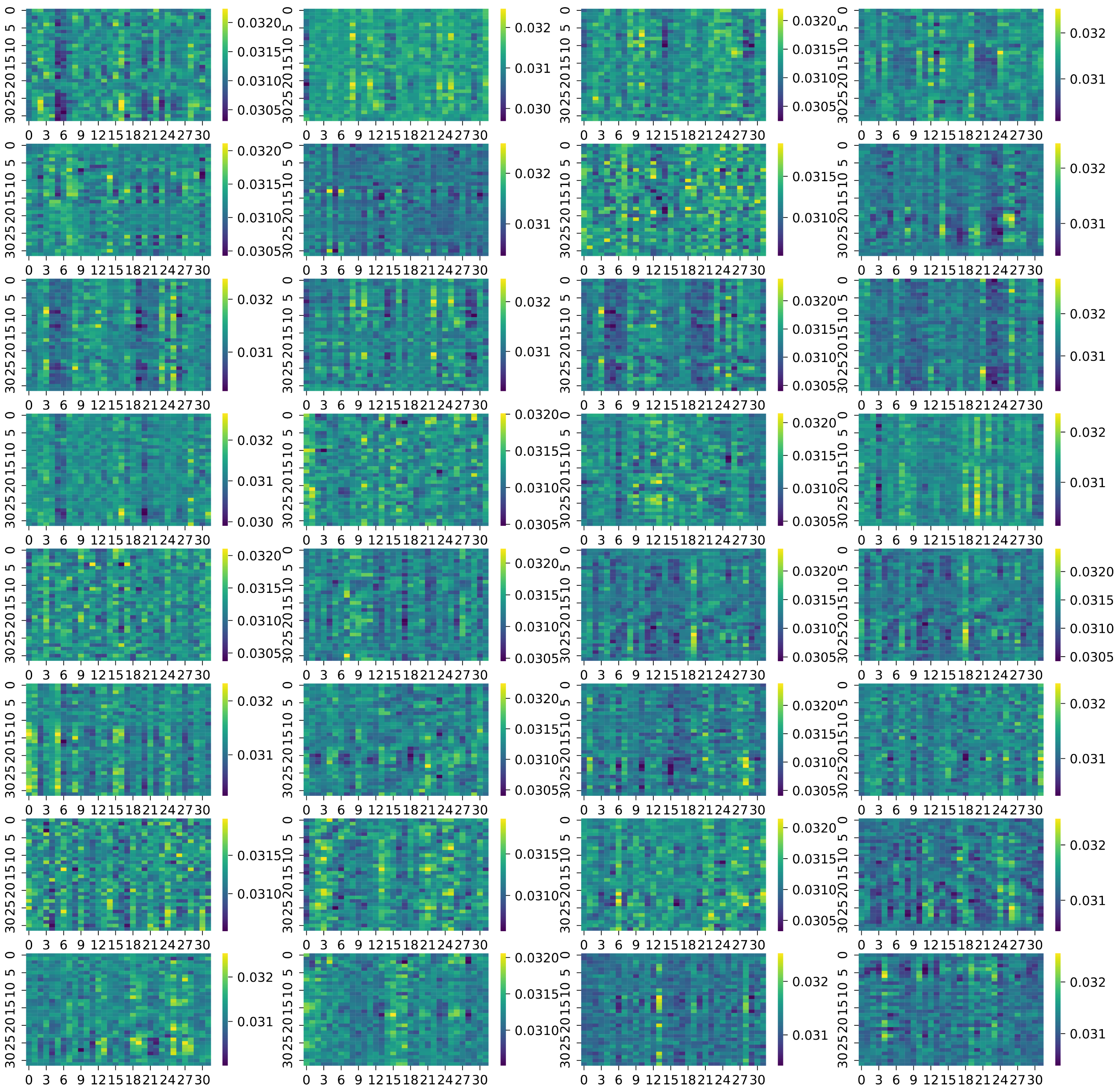

Figure S11. Attention weight distribution of DECENT on cumulus-like reads. Visualization of average head attention weight matrices captured by the multi-head attention module.

A.12 Supplementary Figure S12: Effect of DMR Enrichment on Model Classification Performance.

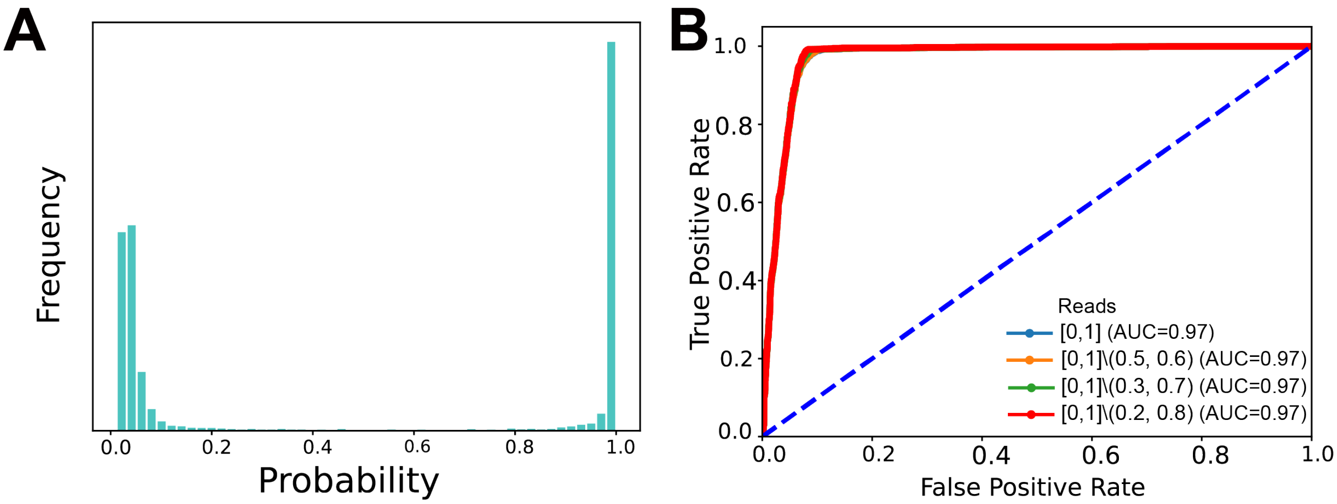

Figure S12. Effect of Differentially Methylated Regions (DMR) Enrichment on Model Classification Performance.

A.13 Supplementary Figure S13: Ablation studies for the CNN, LSTM, and attention components.

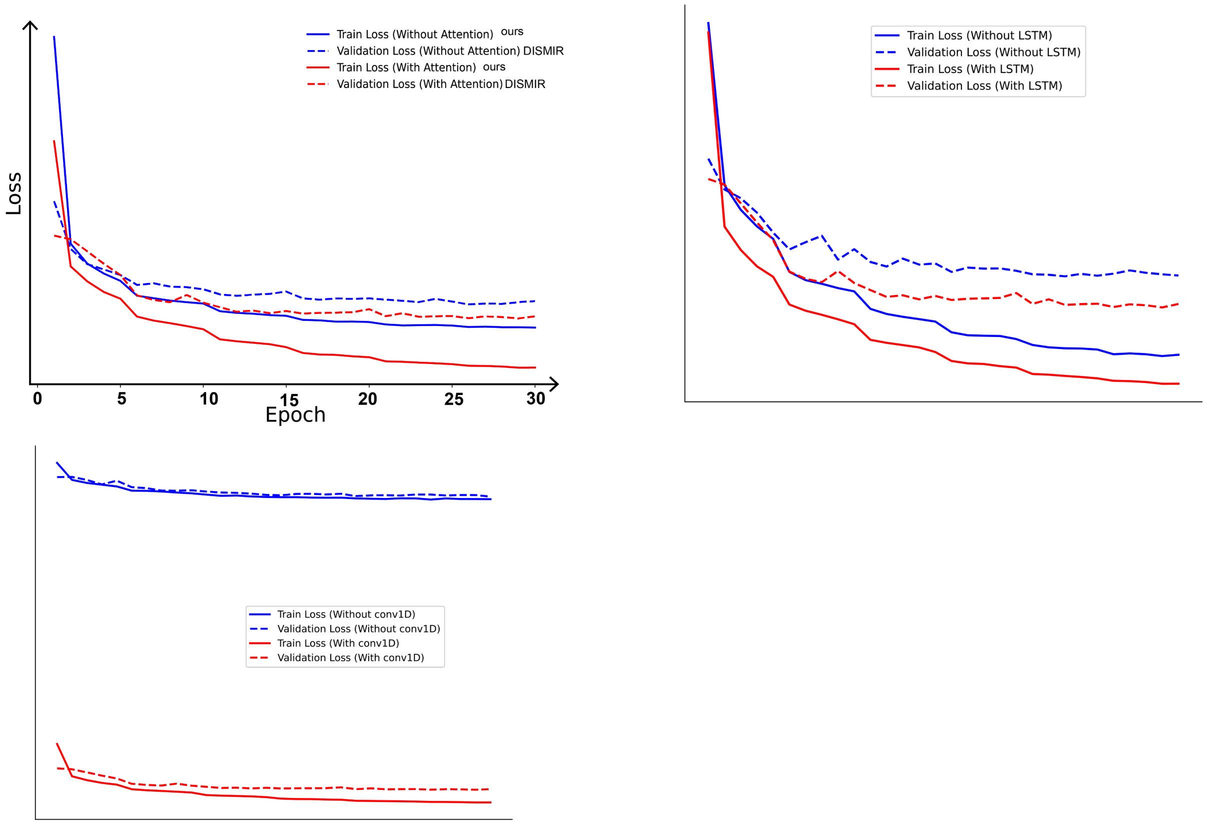

Figure S13. Ablation studies for the CNN, LSTM, and attention components. Training loss and validation loss versus epochs with or without each components.

A.14 Supplementary Figure S14: Proportion of DMR Coverage Across Chromosomes.

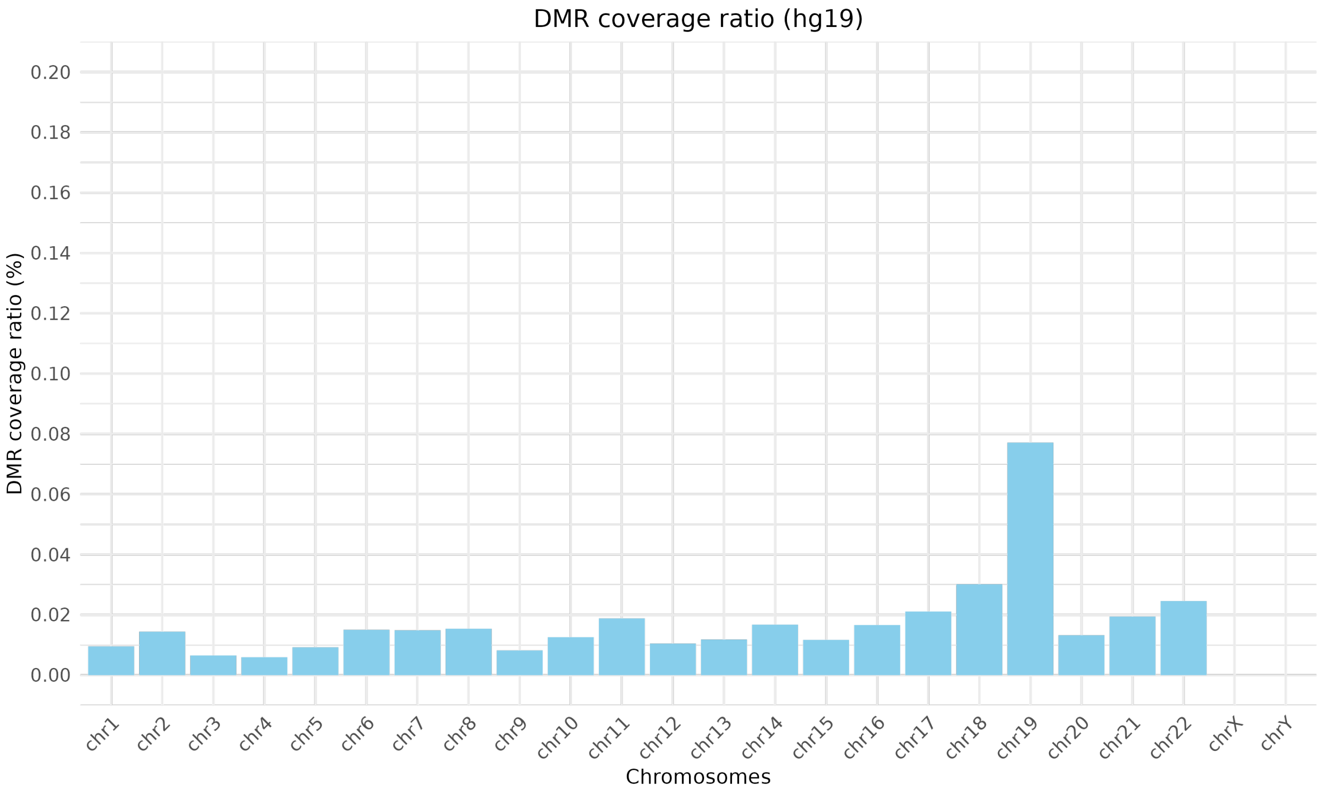

Figure S14. Proportion of DMR Coverage Across Chromosomes.

A.15 Supplementary Figure S15: Analysis Results of DECENT on Moderate Contaminated SECM samples (I)

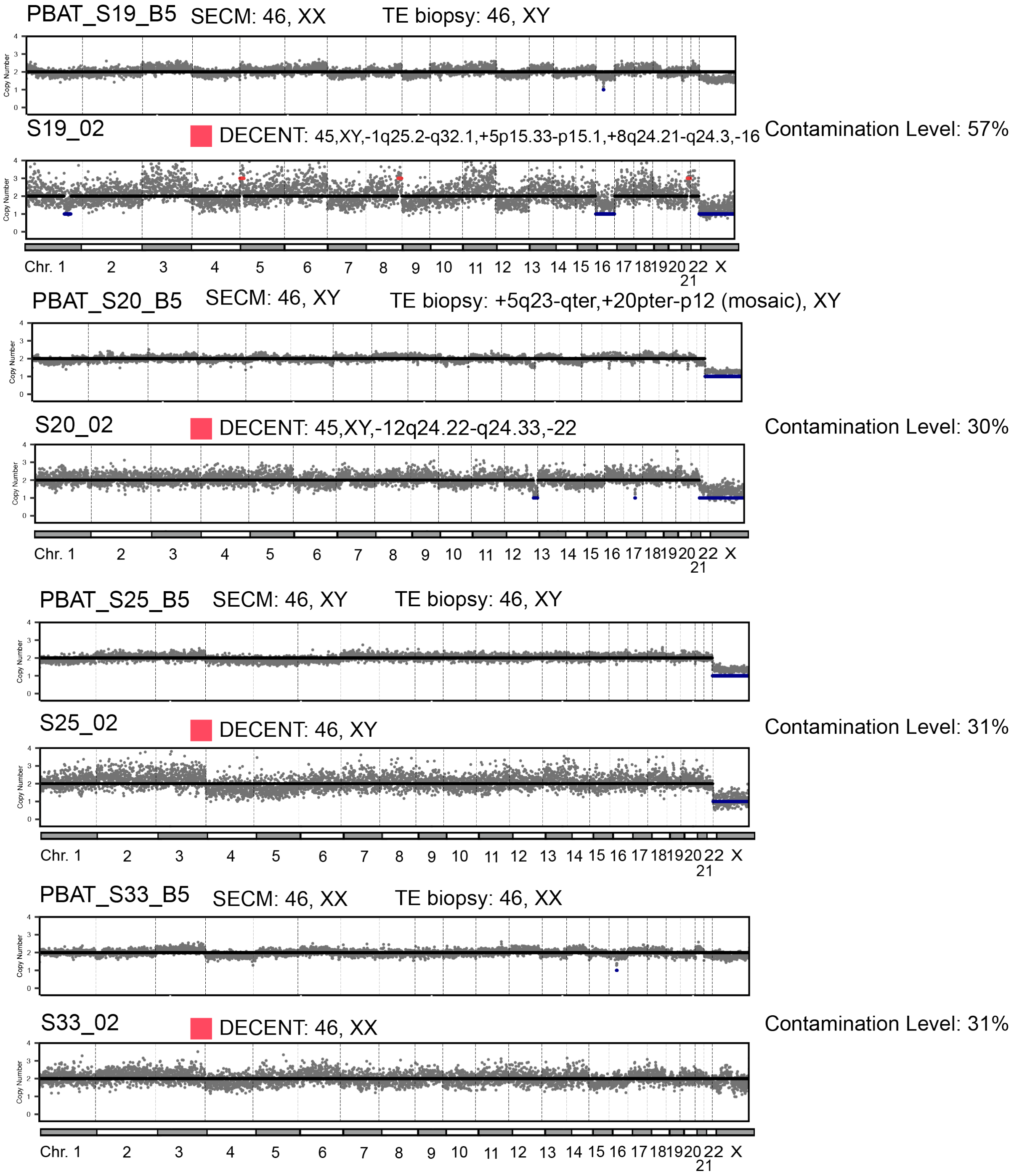

Figure S15. Analysis results of DECENT on moderate contaminated SECM samples (I). Each pair of plots represents a sample set, with the first plot displaying the chromosome copy number results of the original sample and the second plot showing the chromosome copy number results after applying DECENT filtering with a threshold of 0.2. We show the original SECM, TE and the processed DECENT results.

A.16 Supplementary Figure S16: Analysis Results of DECENT on Moderate Contaminated SECM samples (II)

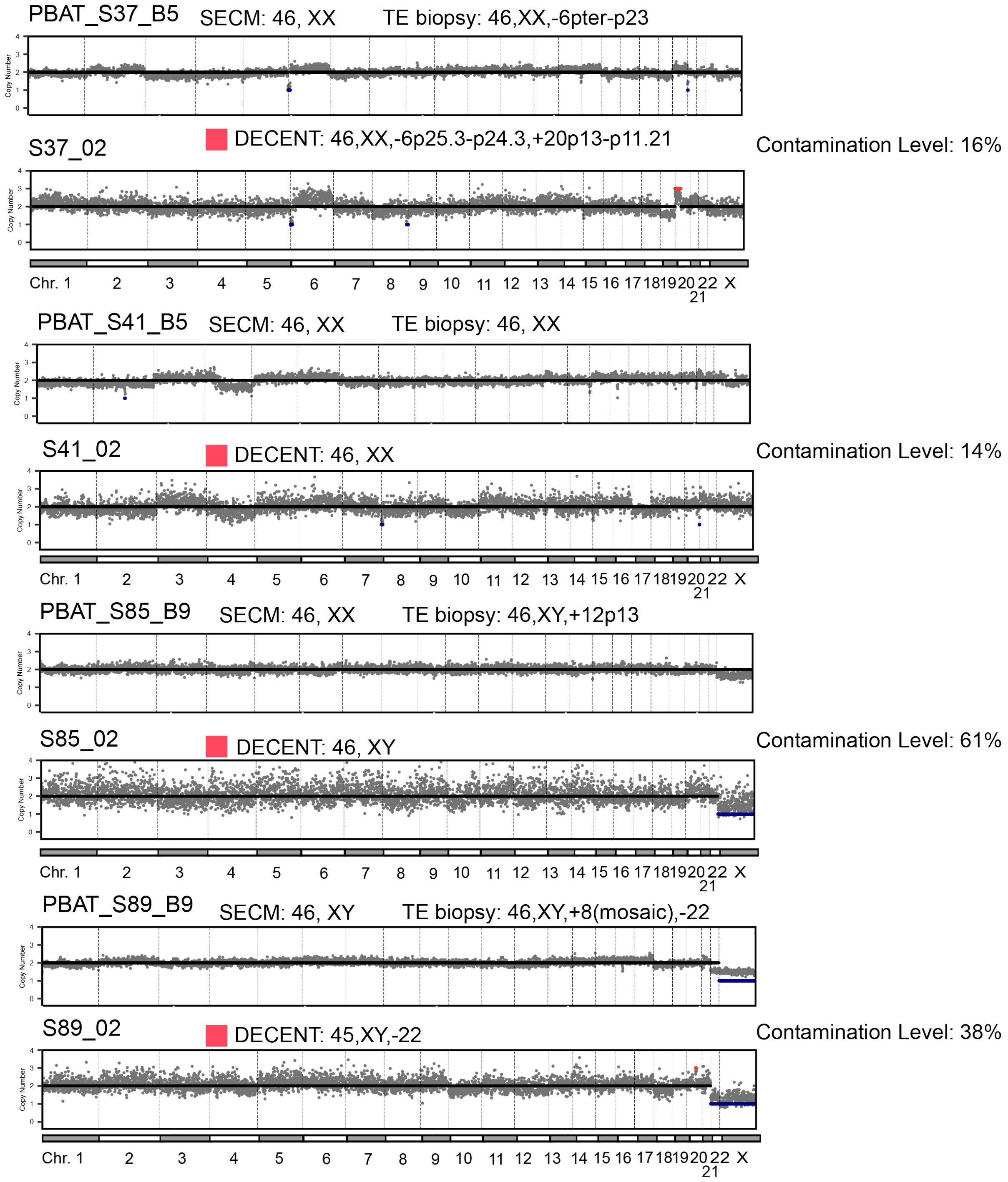

Figure S16. Analysis results of DECENT on moderate contaminated SECM samples (II). Each pair of plots represents a sample set, with the first plot displaying the chromosome copy number results of the original sample and the second plot showing the chromosome copy number results after applying DECENT filtering with a threshold of 0.2. We show the original SECM, TE and the processed DECENT results.

A.17 Supplementary Figure S17: Analysis Results of DECENT on Moderate Contaminated SECM samples (III)

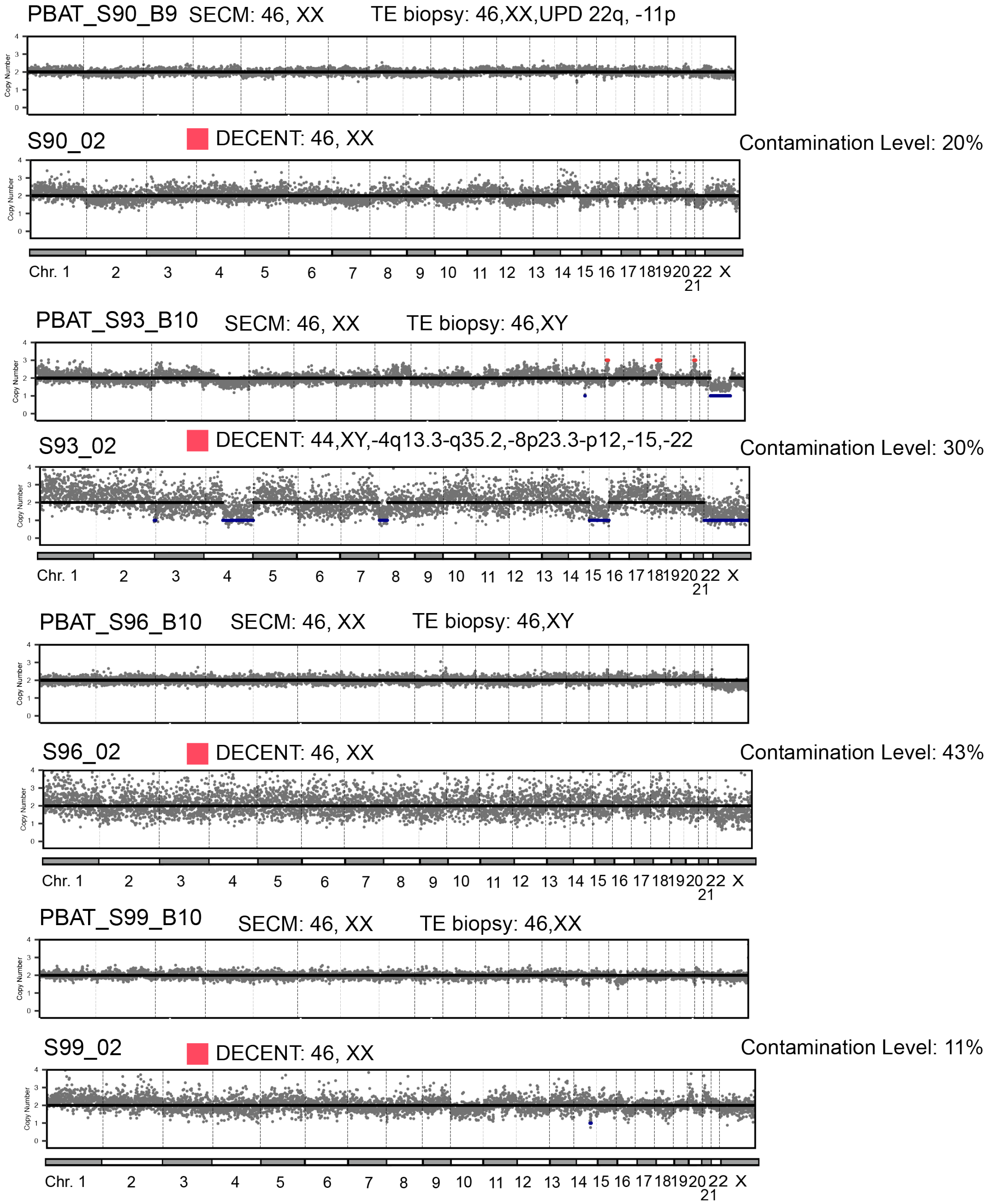

Figure S17. Analysis results of DECENT on moderate contaminated SECM samples (III). Each pair of plots represents a sample set, with the first plot displaying the chromosome copy number results of the original sample and the second plot showing the chromosome copy number results after applying DECENT filtering with a threshold of 0.2. We show the original SECM, TE and the processed DECENT results.

A.18 Supplementary Figure S18: Analysis Results of DECENT on Moderate Contaminated SECM samples (IV)

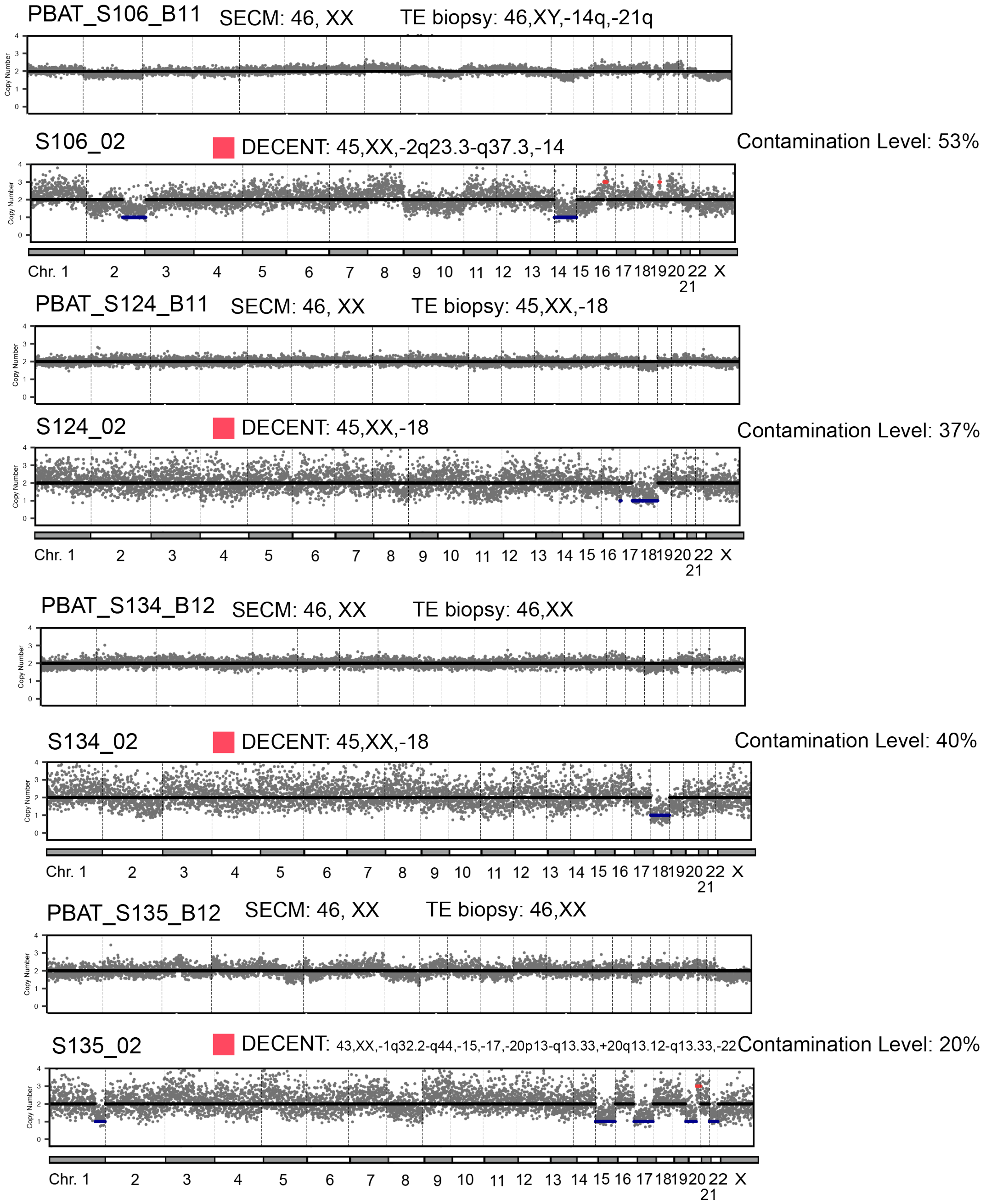

Figure S18. Analysis results of DECENT on moderate contaminated SECM samples (IV). Each pair of plots represents a sample set, with the first plot displaying the chromosome copy number results of the original sample and the second plot showing the chromosome copy number results after applying DECENT filtering with a threshold of 0.2. We show the original SECM, TE and the processed DECENT results.

A.19 Supplementary Figure S19: Analysis Results of DECENT on Moderate Contaminated SECM samples (V)

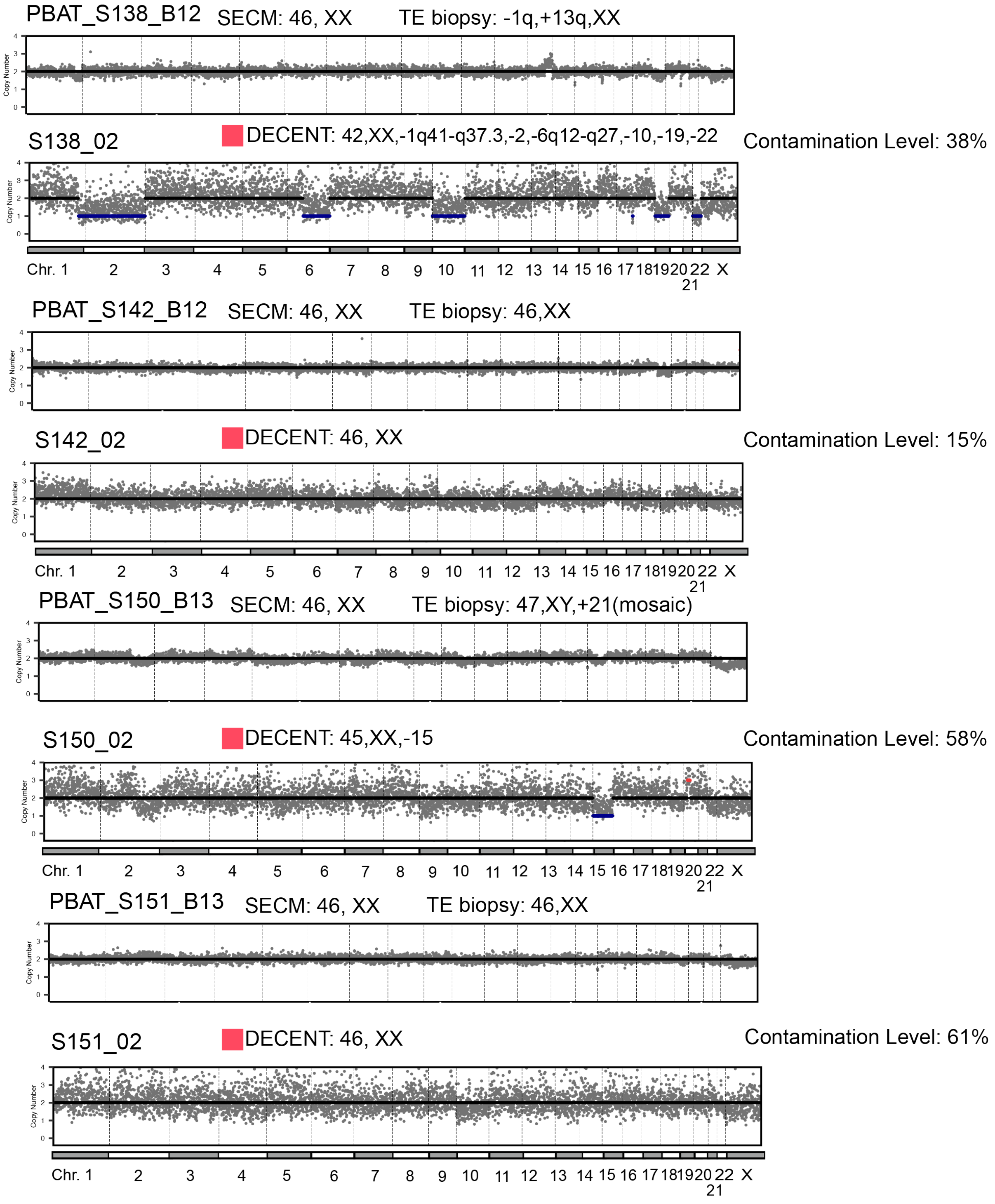

Figure S19. Analysis results of DECENT on moderate contaminated SECM samples (V). Each pair of plots represents a sample set, with the first plot displaying the chromosome copy number results of the original sample and the second plot showing the chromosome copy number results after applying DECENT filtering with a threshold of 0.2. We show the original SECM, TE and the processed DECENT results.

A.20 Supplementary Figure S20: Analysis Results of DECENT on Moderate Contaminated SECM samples (VI)

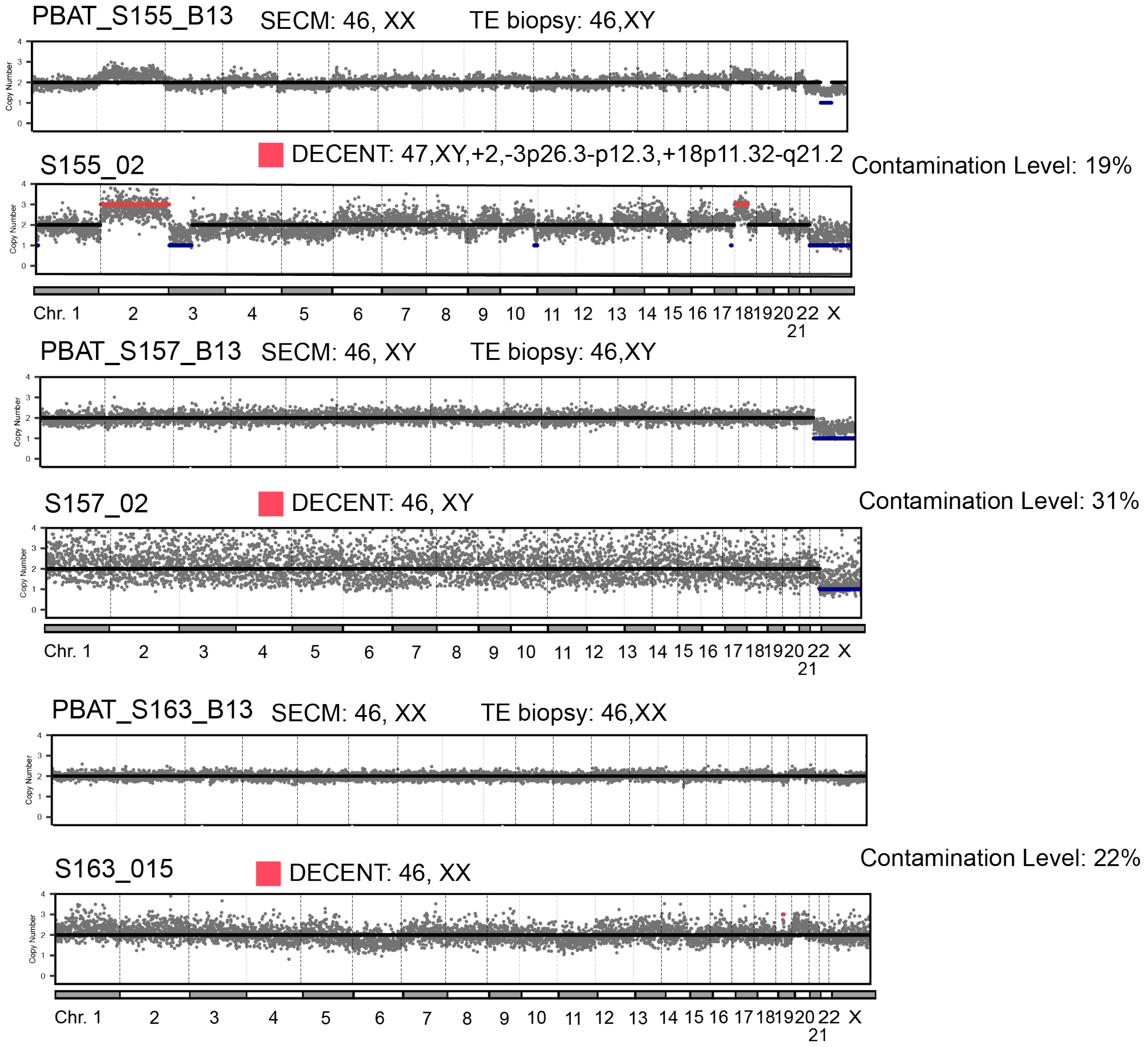

Figure S20. Analysis results of DECENT on moderate contaminated SECM samples (VI). Each pair of plots represents a sample set, with the first plot displaying the chromosome copy number results of the original sample and the second plot showing the chromosome copy number results after applying DECENT filtering with a threshold of 0.2. We show the original SECM, TE and the processed DECENT results.

A.21 Supplementary Figure S21: Analysis Results of DECENT on Moderate Contaminated SECM samples (VII)

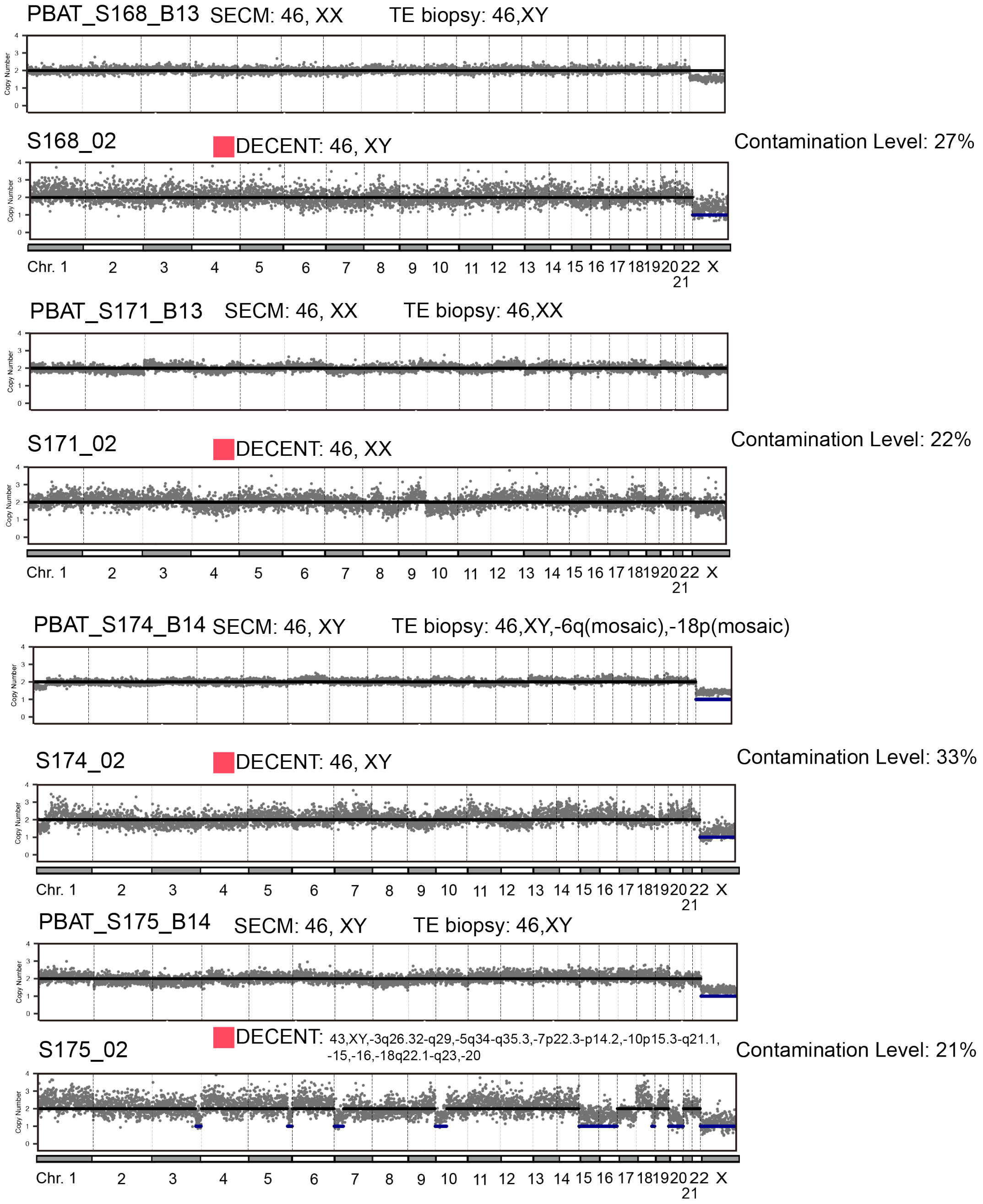

Figure S21. Analysis results of DECENT on moderate contaminated SECM samples (VII). Each pair of plots represents a sample set, with the first plot displaying the chromosome copy number results of the original sample and the second plot showing the chromosome copy number results after applying DECENT filtering with a threshold of 0.2. We show the original SECM, TE and the processed DECENT results.

A.22 Supplementary Figure S22: Analysis Results of DECENT on Moderate Contaminated SECM samples (VIII)

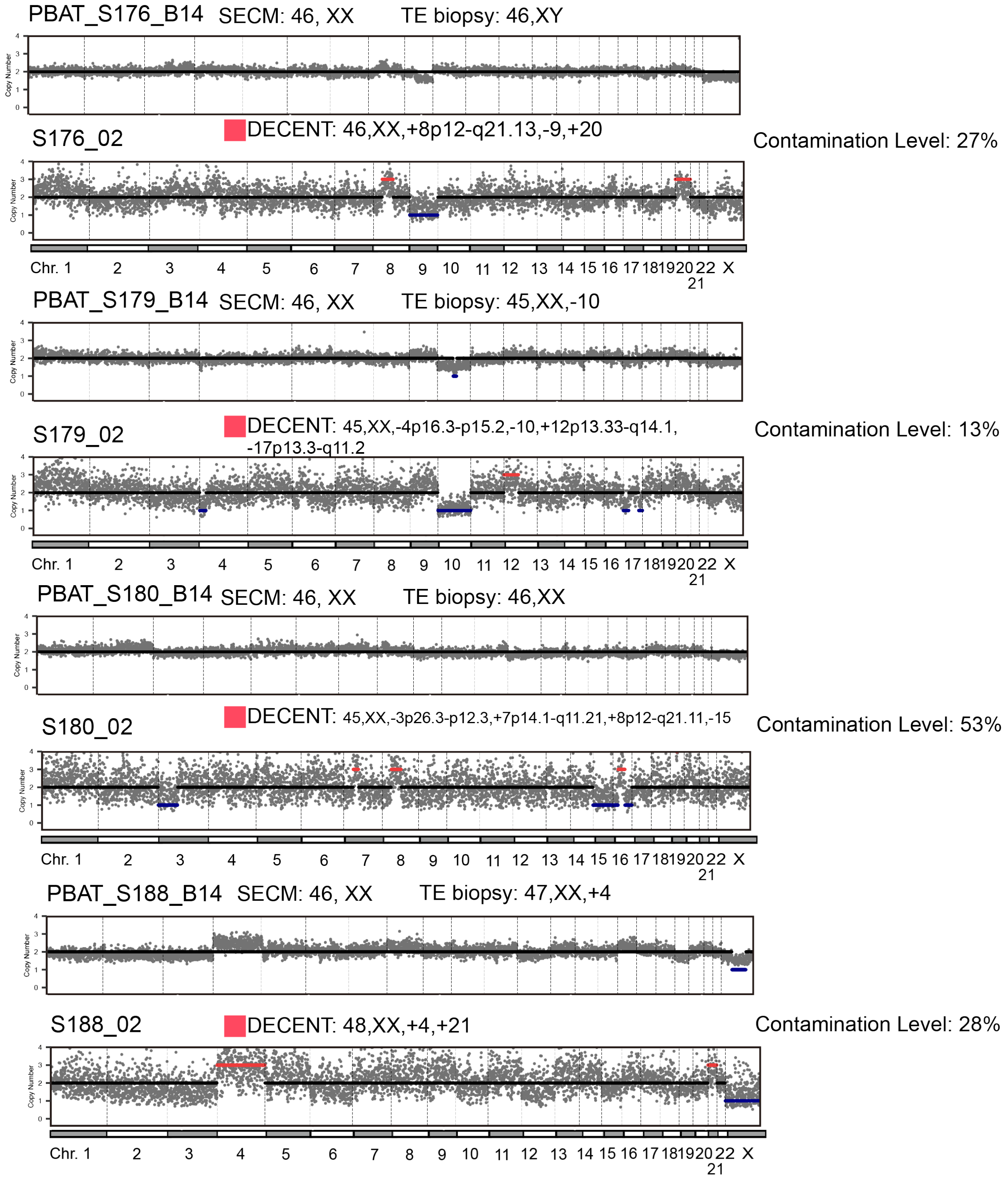

Figure S22. Analysis results of DECENT on moderate contaminated SECM samples (VIII). Each pair of plots represents a sample set, with the first plot displaying the chromosome copy number results of the original sample and the second plot showing the chromosome copy number results after applying DECENT filtering with a threshold of 0.2. We show the original SECM, TE and the processed DECENT results.

A.23 Supplementary Figure S23: Analysis Results of DECENT on Moderate Contaminated SECM samples (IX)

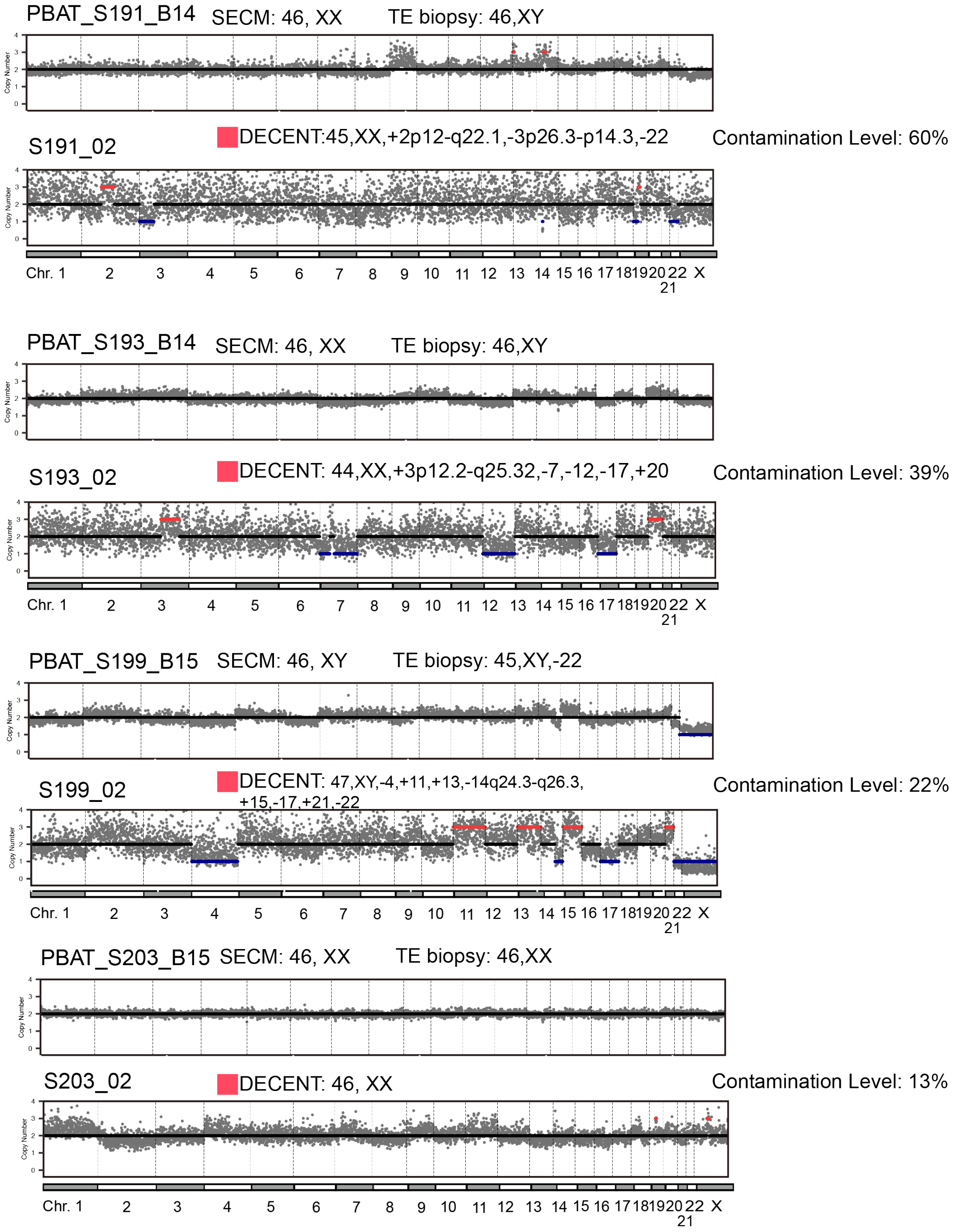

Figure S23. Analysis results of DECENT on moderate contaminated SECM samples (IX). Each pair of plots represents a sample set, with the first plot displaying the chromosome copy number results of the original sample and the second plot showing the chromosome copy number results after applying DECENT filtering with a threshold of 0.2. We show the original SECM, TE and the processed DECENT results.

A.24 Supplementary Figure S24: Analysis Results of DECENT on Moderate Contaminated SECM samples (X)

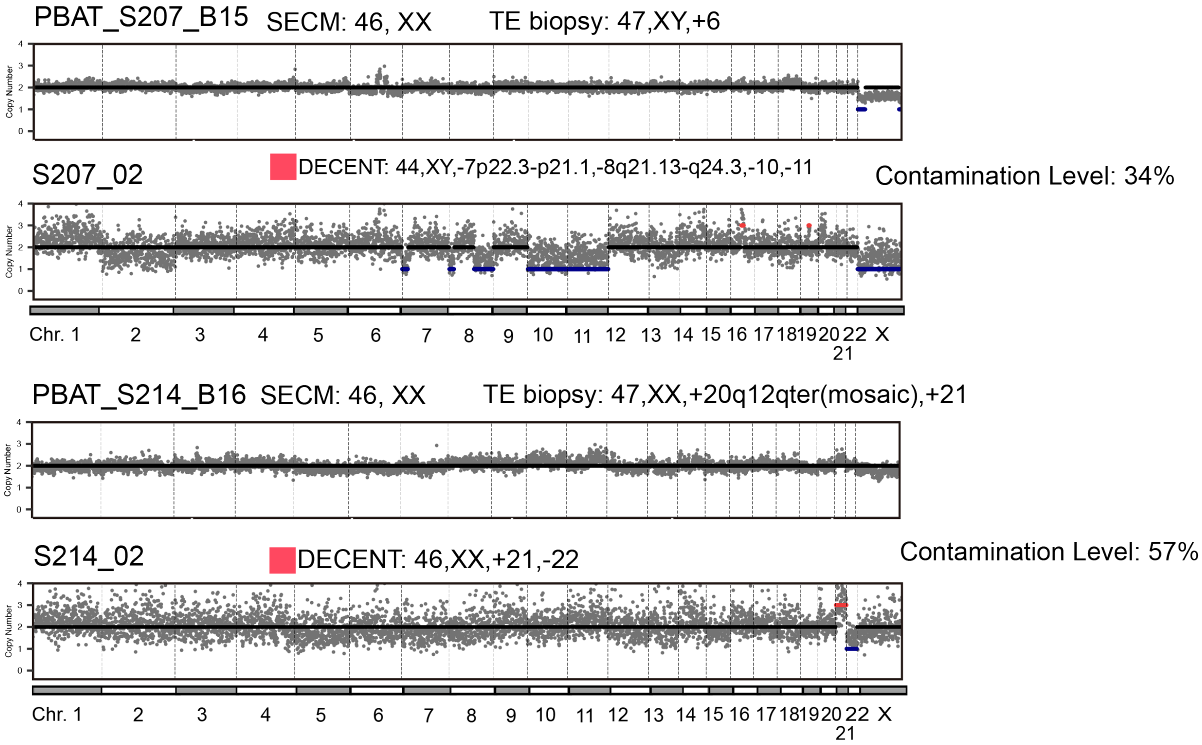

Figure S24. Analysis results of DECENT on moderate contaminated SECM samples (X). Each pair of plots represents a sample set, with the first plot displaying the chromosome copy number results of the original sample and the second plot showing the chromosome copy number results after applying DECENT filtering with a threshold of 0.2. We show the original SECM, TE and the processed DECENT results.

A.25 Supplementary Figure S25: Analysis Results of DECENT on Severe Contaminated SECM samples (I)

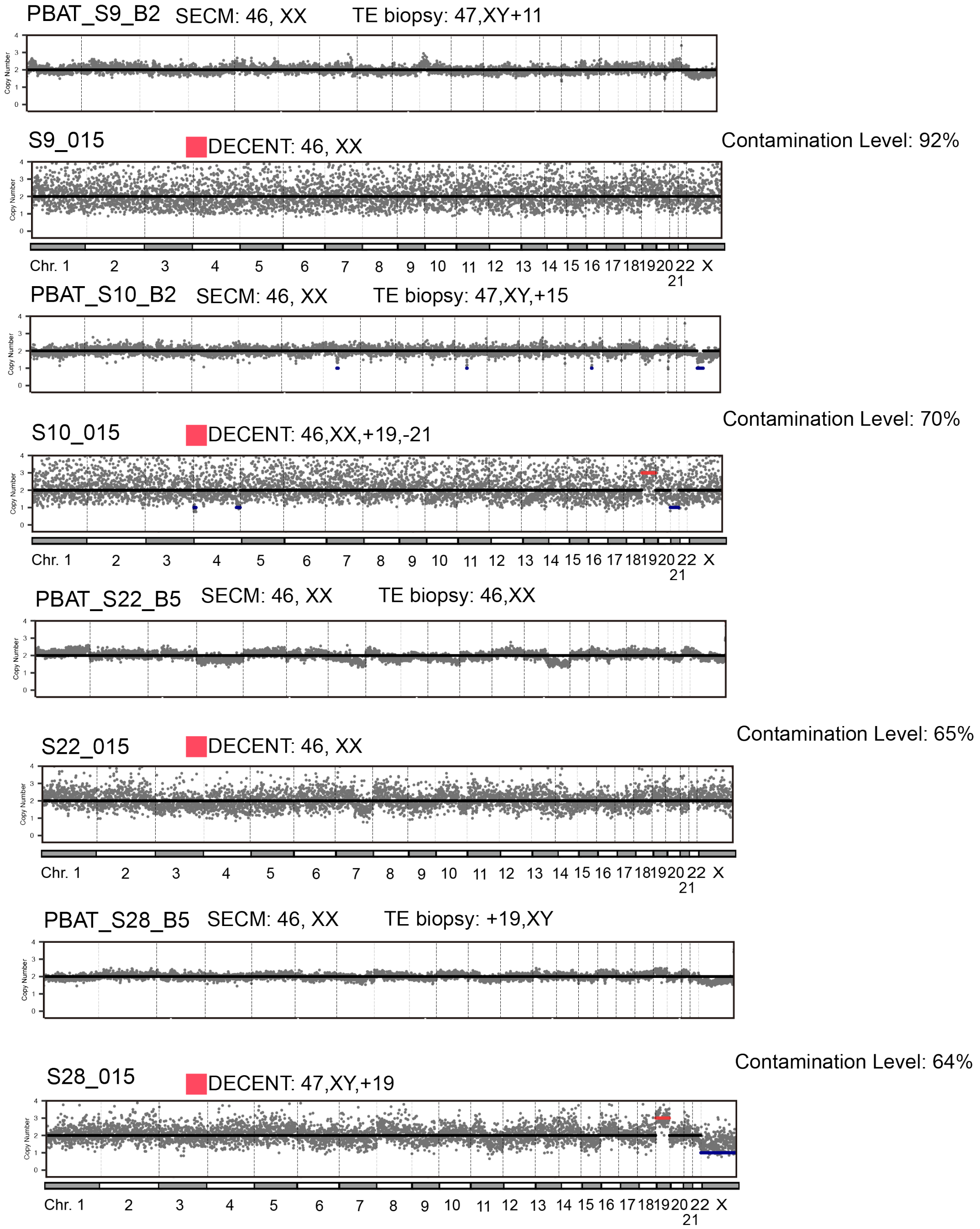

Figure S25. Analysis results of DECENT on severe contaminated SECM samples (I). Each pair of plots represents a sample set, with the first plot displaying the chromosome copy number results of the original sample and the second plot showing the chromosome copy number results after applying DECENT filtering with a threshold of 0.15. We show the original SECM, TE and the processed DECENT results.

A.26 Supplementary Figure S26: Analysis Results of DECENT on Severe Contaminated SECM samples (II)

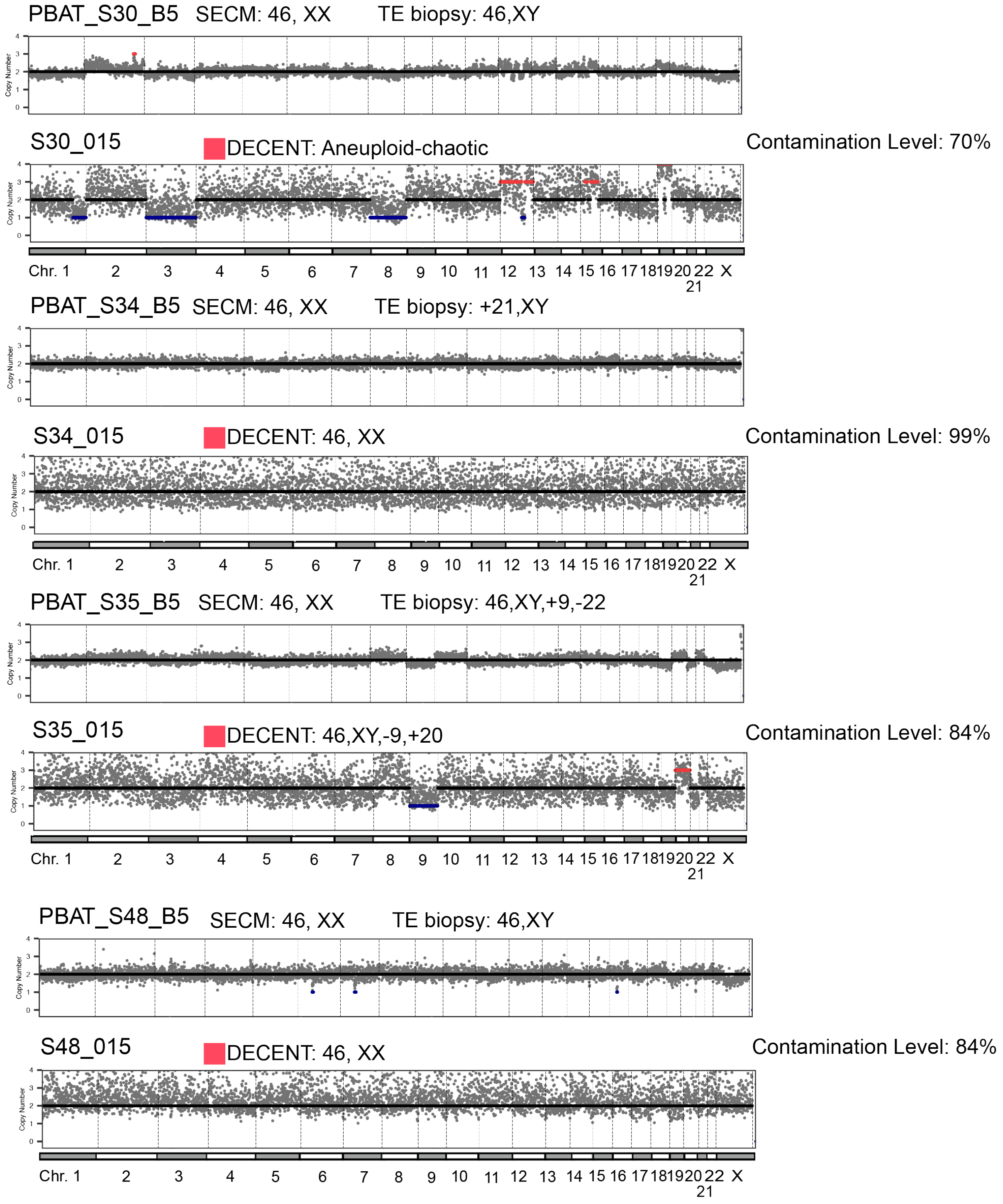

Figure S26. Analysis results of DECENT on severe contaminated SECM samples (II). Each pair of plots represents a sample set, with the first plot displaying the chromosome copy number results of the original sample and the second plot showing the chromosome copy number results after applying DECENT filtering with a threshold of 0.15. We show the original SECM, TE and the processed DECENT results.

A.27 Supplementary Figure S27: Analysis Results of DECENT on Severe Contaminated SECM samples (III)

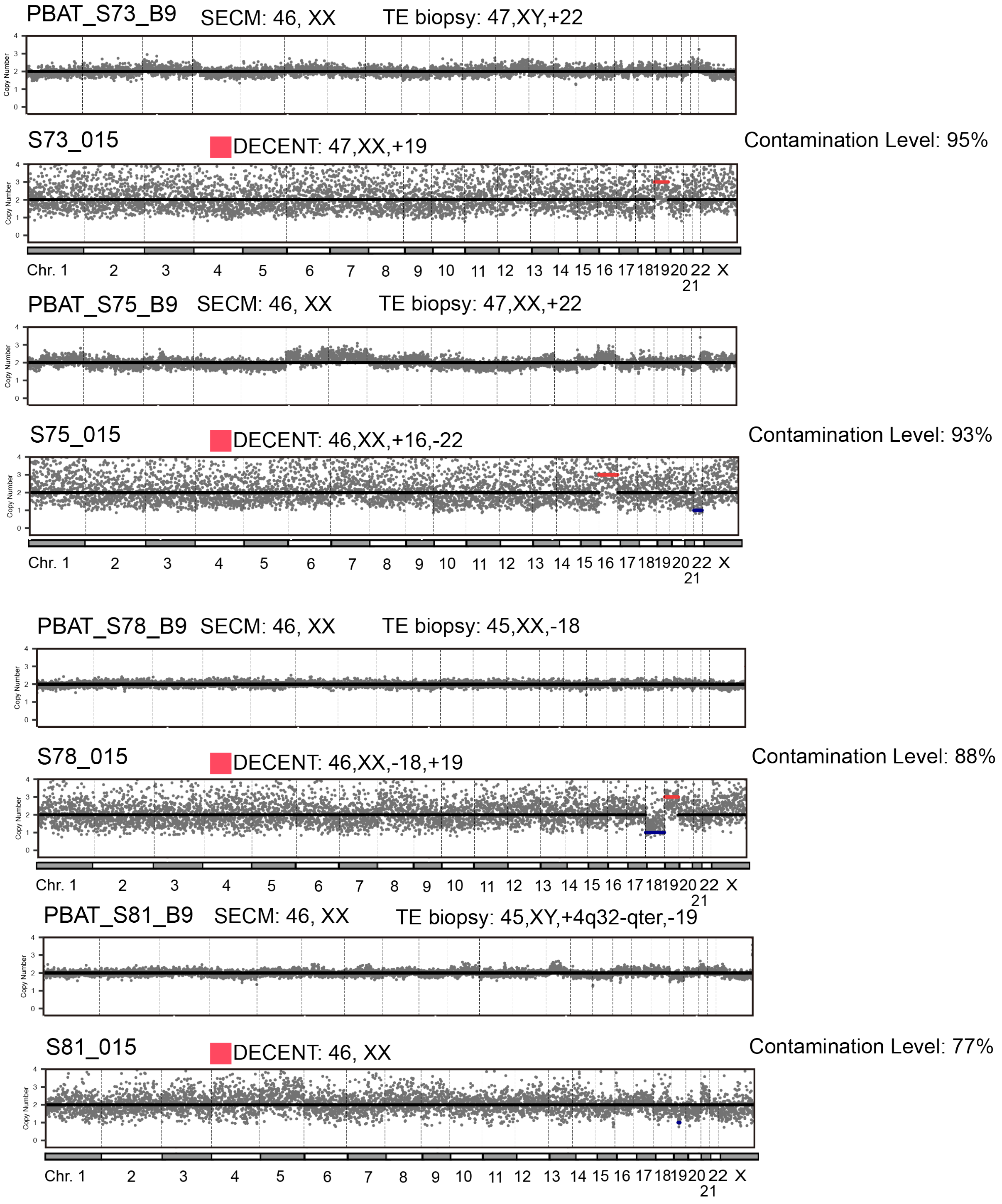

Figure S27. Analysis results of DECENT on severe contaminated SECM samples (III). Each pair of plots represents a sample set, with the first plot displaying the chromosome copy number results of the original sample and the second plot showing the chromosome copy number results after applying DECENT filtering with a threshold of 0.15. We show the original SECM, TE and the processed DECENT results.

A.28 Supplementary Figure S28: Analysis Results of DECENT on Severe Contaminated SECM samples (IV)

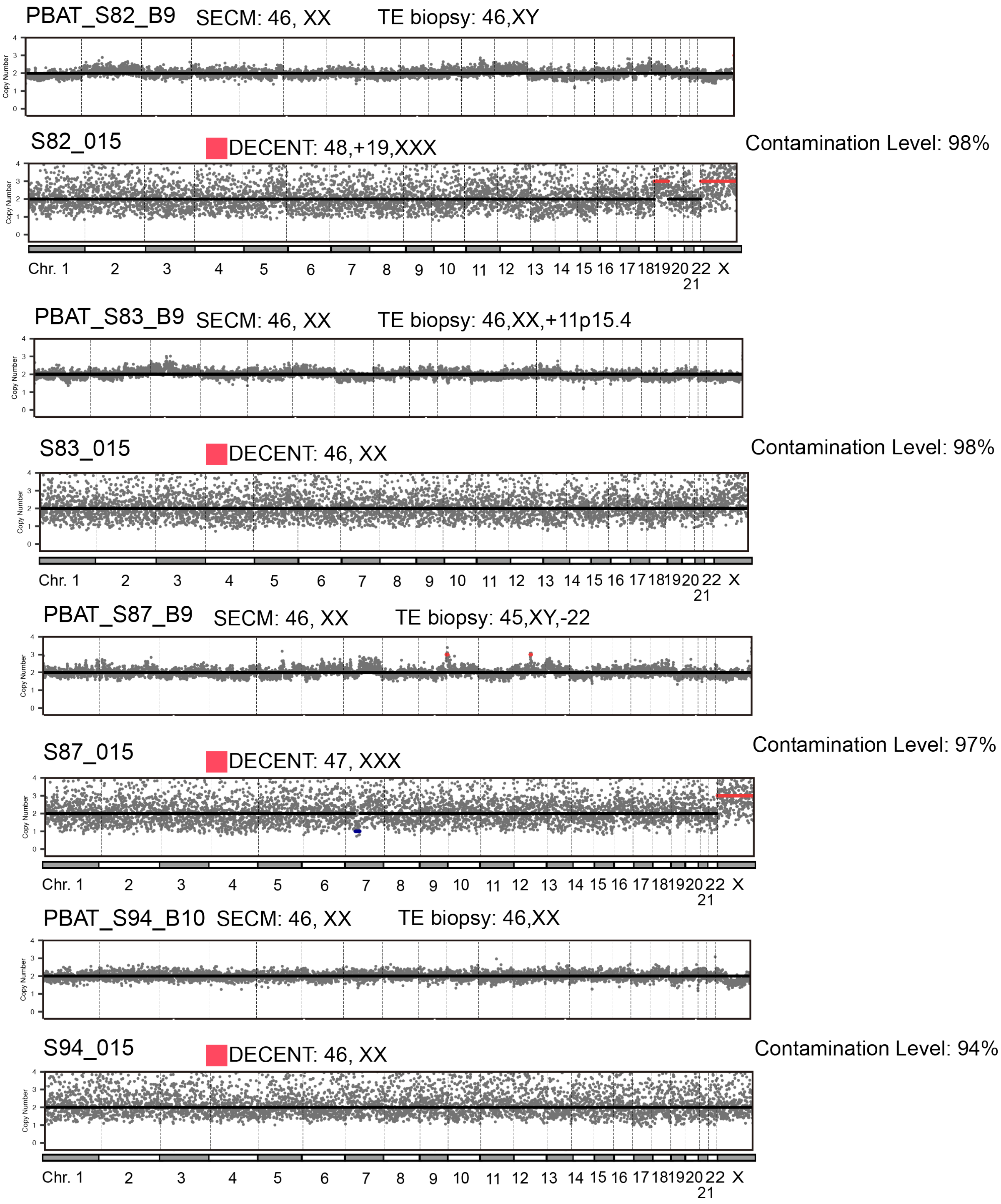

Figure S28. Analysis results of DECENT on severe contaminated SECM samples (IV). Each pair of plots represents a sample set, with the first plot displaying the chromosome copy number results of the original sample and the second plot showing the chromosome copy number results after applying DECENT filtering with a threshold of 0.15. We show the original SECM, TE and the processed DECENT results.

A.29 Supplementary Figure S29: Analysis Results of DECENT on Severe Contaminated SECM samples (V)

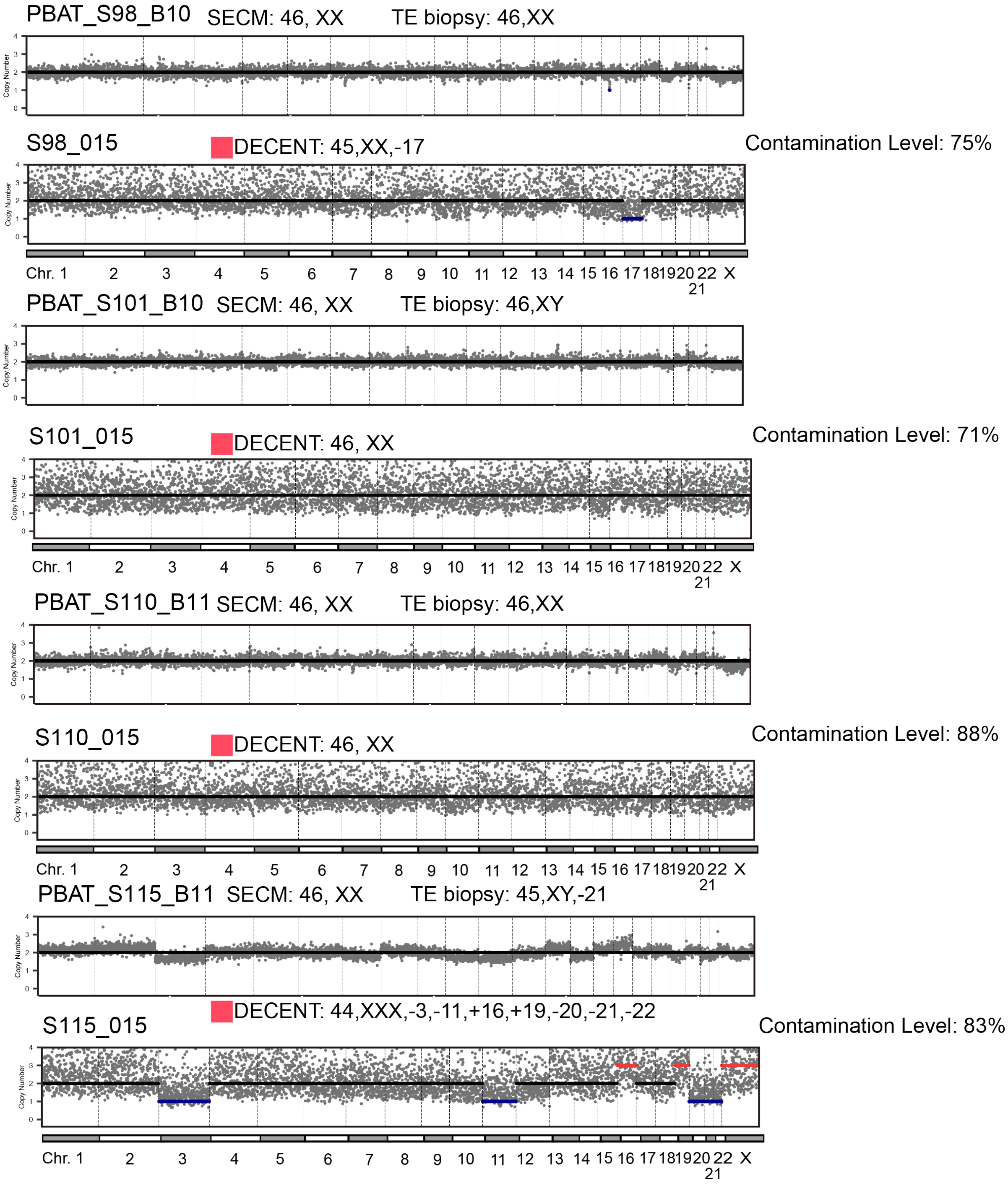

Figure S29. Analysis results of DECENT on severe contaminated SECM samples (V). Each pair of plots represents a sample set, with the first plot displaying the chromosome copy number results of the original sample and the second plot showing the chromosome copy number results after applying DECENT filtering with a threshold of 0.15. We show the original SECM, TE and the processed DECENT results.

A.30 Supplementary Figure S30: Analysis Results of DECENT on Severe Contaminated SECM samples (VI)

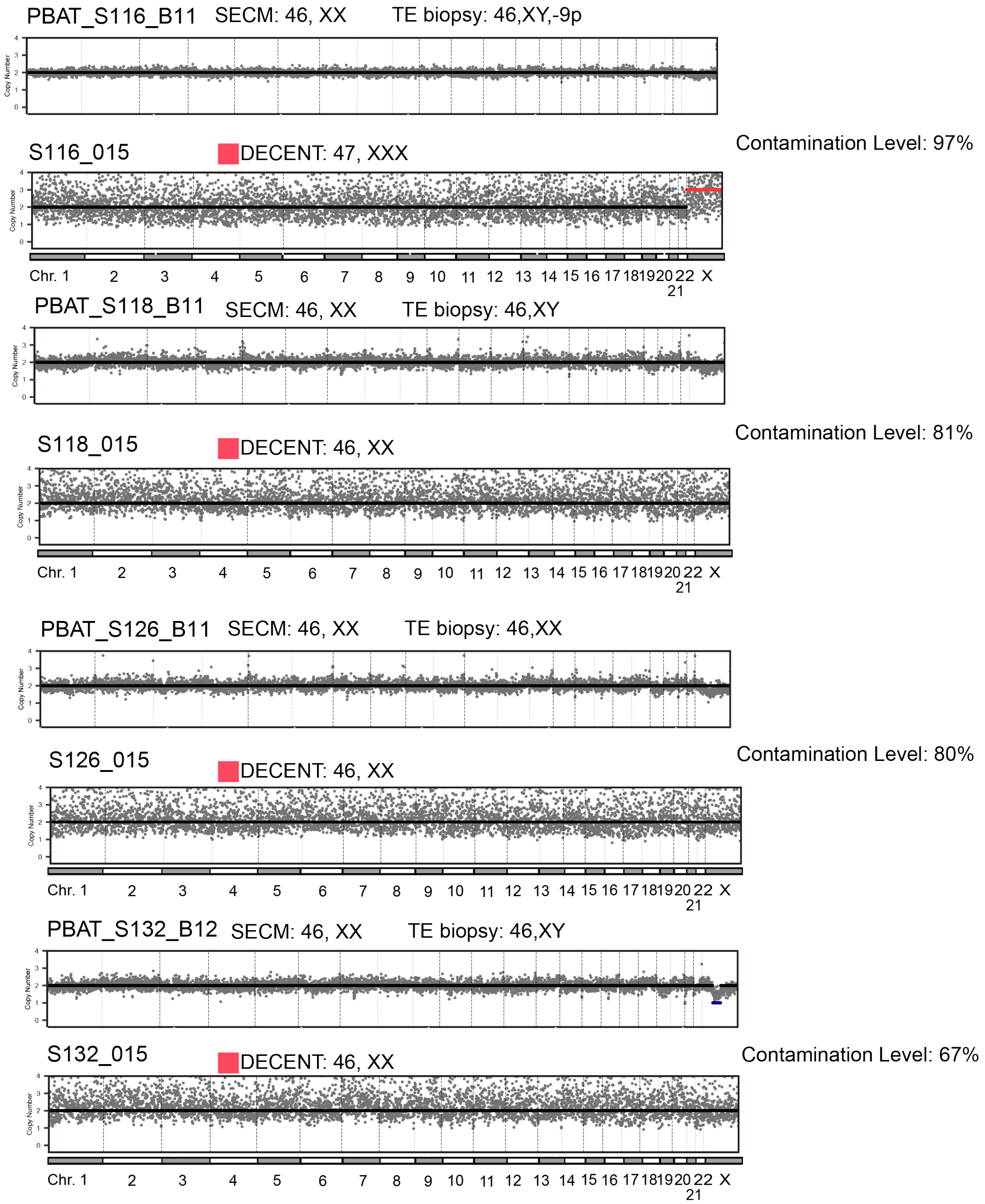

Figure S30. Analysis results of DECENT on severe contaminated SECM samples (VI). Each pair of plots represents a sample set, with the first plot displaying the chromosome copy number results of the original sample and the second plot showing the chromosome copy number results after applying DECENT filtering with a threshold of 0.15. We show the original SECM, TE and the processed DECENT results.

A.31 Supplementary Figure S31: Analysis Results of DECENT on Severe Contaminated SECM samples (VII)

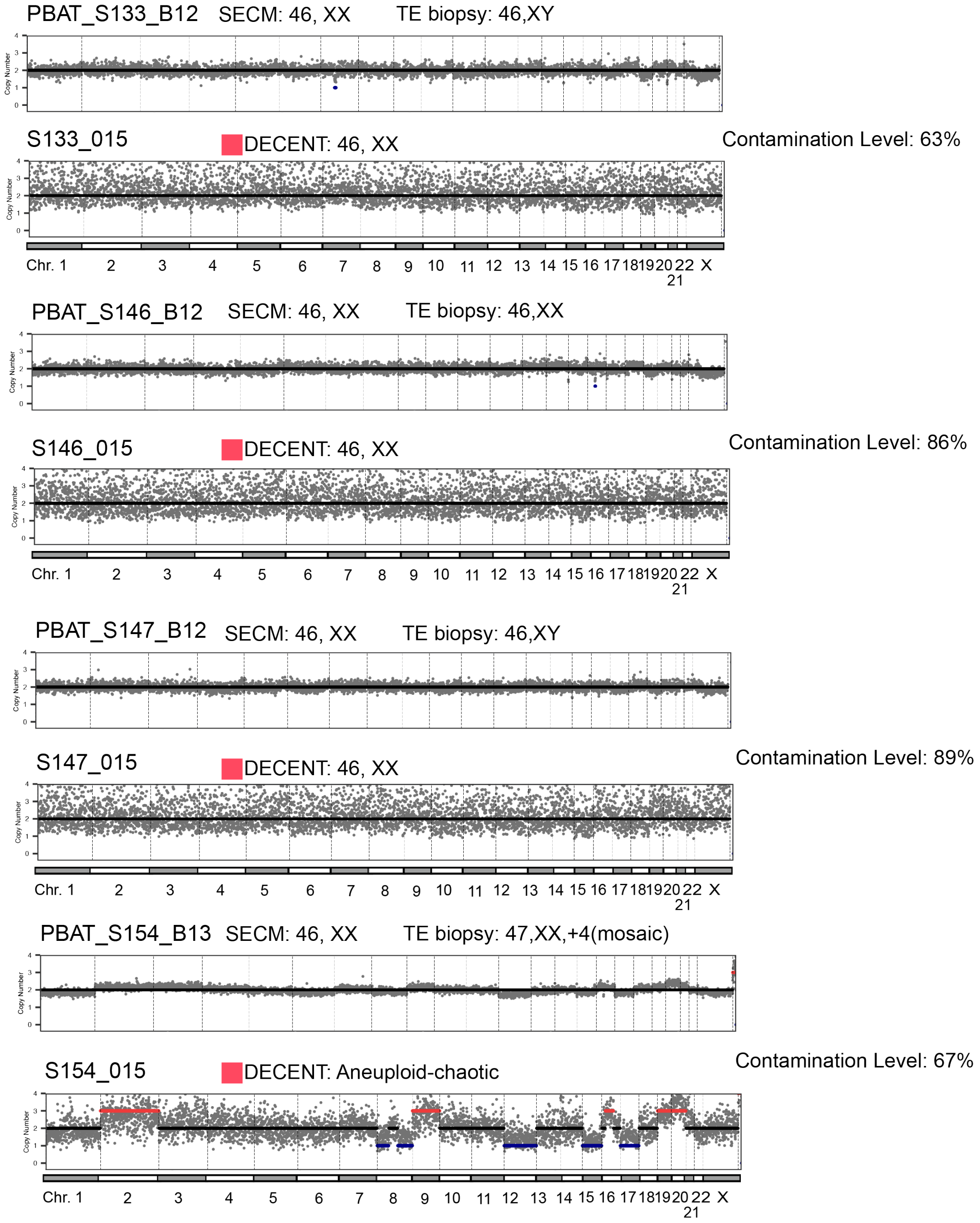

Figure S31. Analysis results of DECENT on severe contaminated SECM samples (VII). Each pair of plots represents a sample set, with the first plot displaying the chromosome copy number results of the original sample and the second plot showing the chromosome copy number results after applying DECENT filtering with a threshold of 0.15. We show the original SECM, TE and the processed DECENT results.

A.32 Supplementary Figure S32: Analysis Results of DECENT on Severe Contaminated SECM samples (VIII)

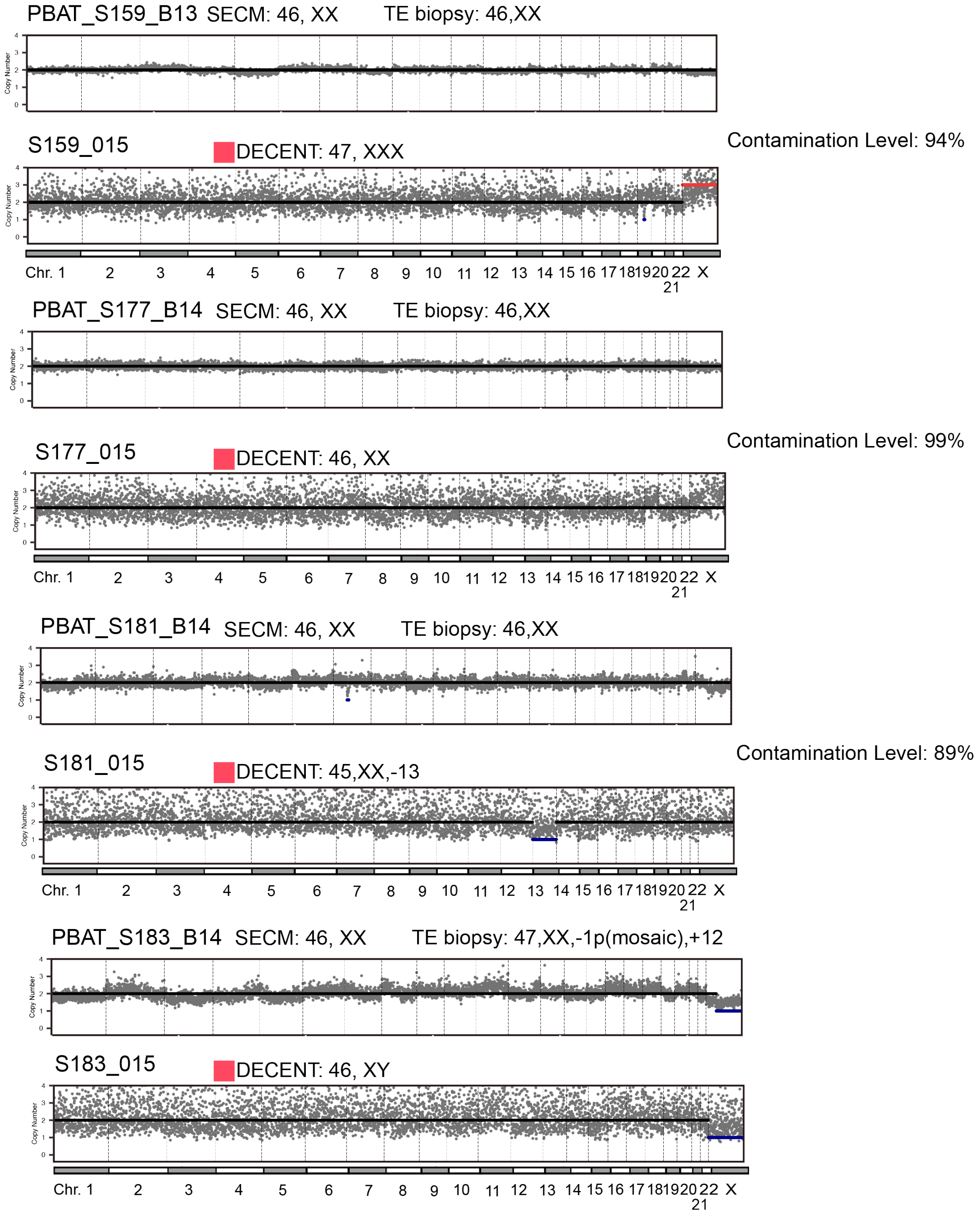

Figure S32. Analysis results of DECENT on severe contaminated SECM samples (VIII). Each pair of plots represents a sample set, with the first plot displaying the chromosome copy number results of the original sample and the second plot showing the chromosome copy number results after applying DECENT filtering with a threshold of 0.15. We show the original SECM, TE and the processed DECENT results.

A.33 Supplementary Figure S33: Analysis Results of DECENT on Severe Contaminated SECM samples (IX)

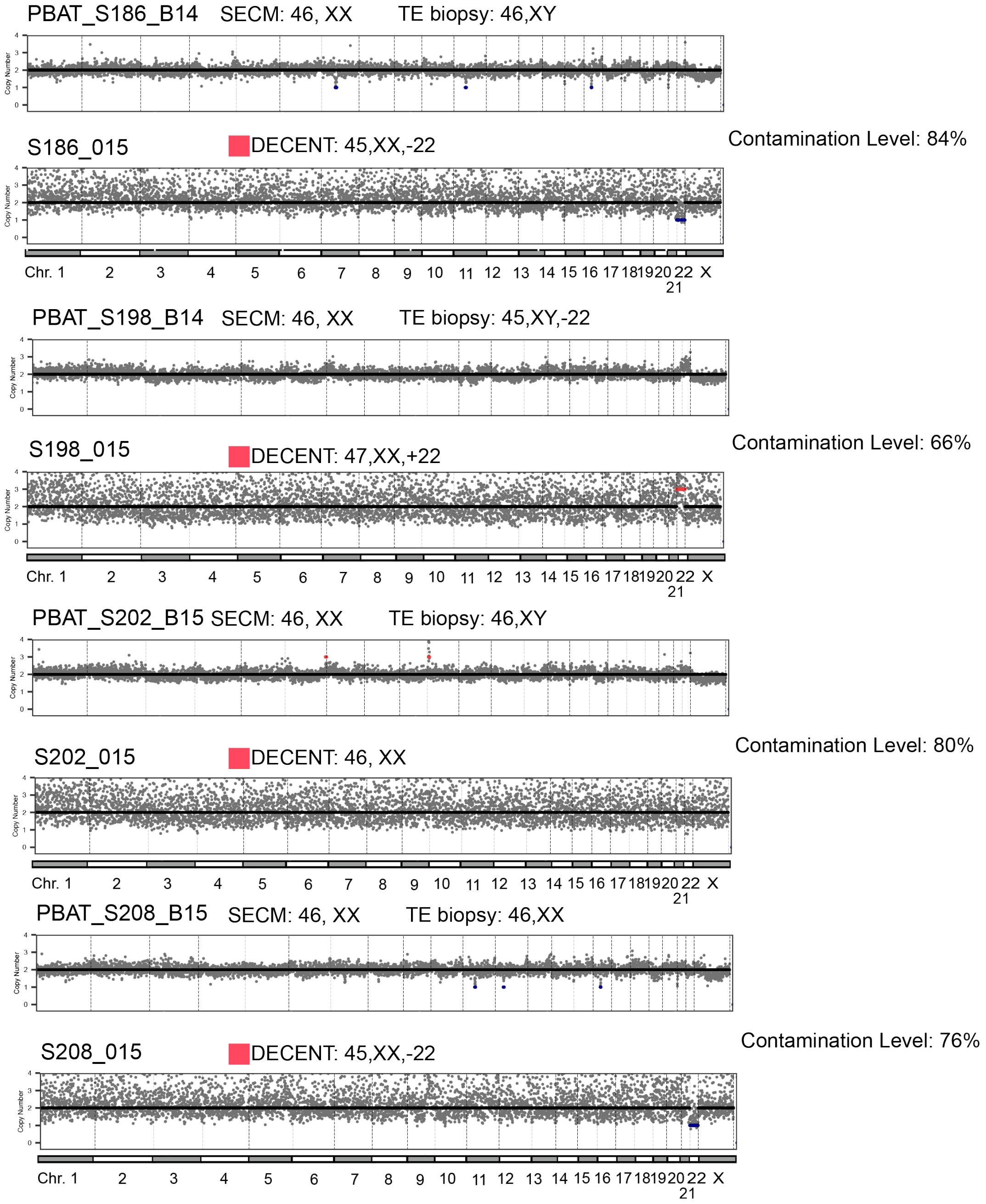

Figure S33. Analysis results of DECENT on severe contaminated SECM samples (IX). Each pair of plots represents a sample set, with the first plot displaying the chromosome copy number results of the original sample and the second plot showing the chromosome copy number results after applying DECENT filtering with a threshold of 0.15. We show the original SECM, TE and the processed DECENT results.

A.34 Supplementary Figure S34: Analysis Results of DECENT on Severe Contaminated SECM samples (X)

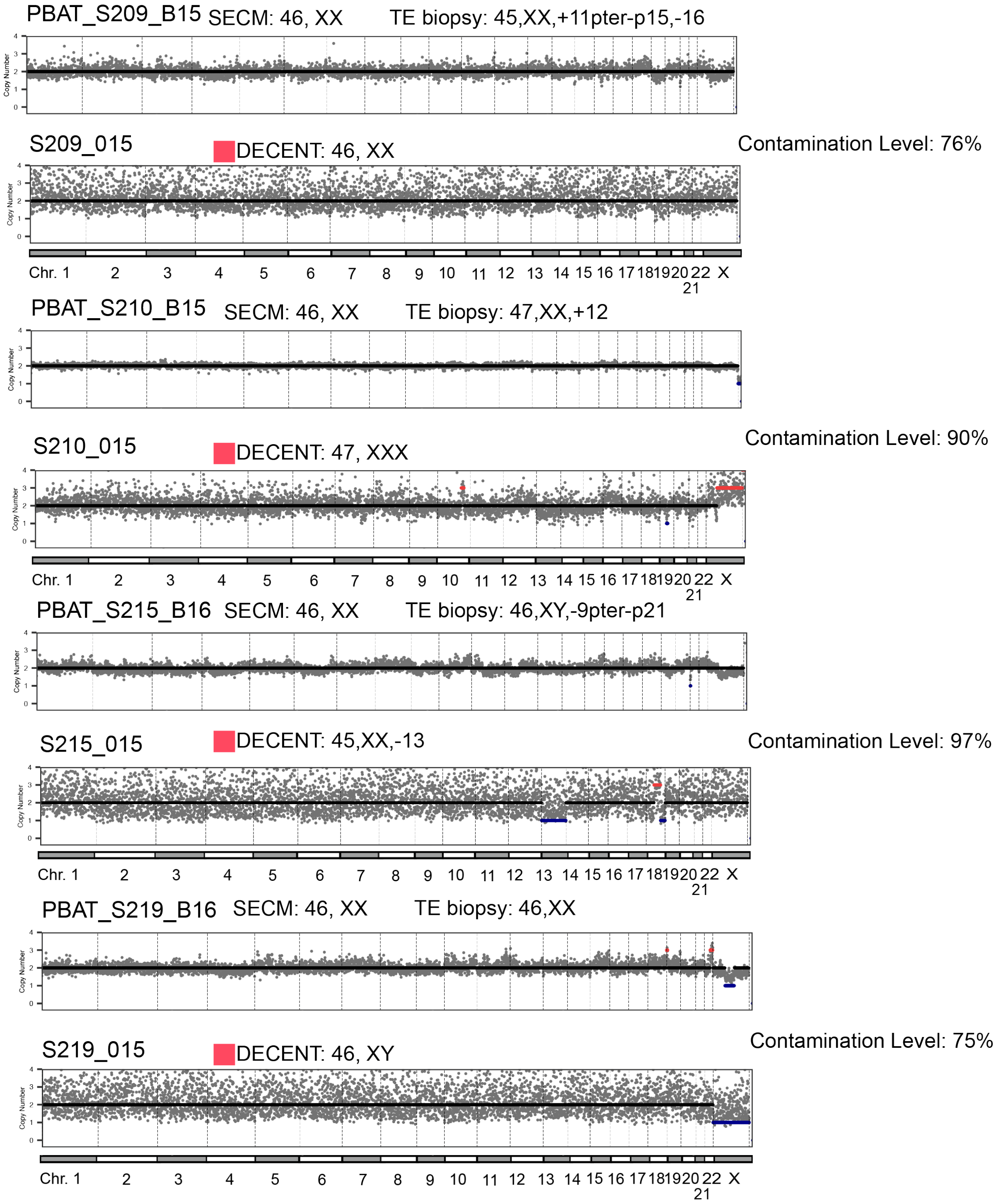

Figure S34. Analysis results of DECENT on severe contaminated SECM samples (X). Each pair of plots represents a sample set, with the first plot displaying the chromosome copy number results of the original sample and the second plot showing the chromosome copy number results after applying DECENT filtering with a threshold of 0.15. We show the original SECM, TE and the processed DECENT results.

A.35 Supplementary Figure S35: Enrichment analysis using ChIP-seq data from ICM cells and MII oocytes.

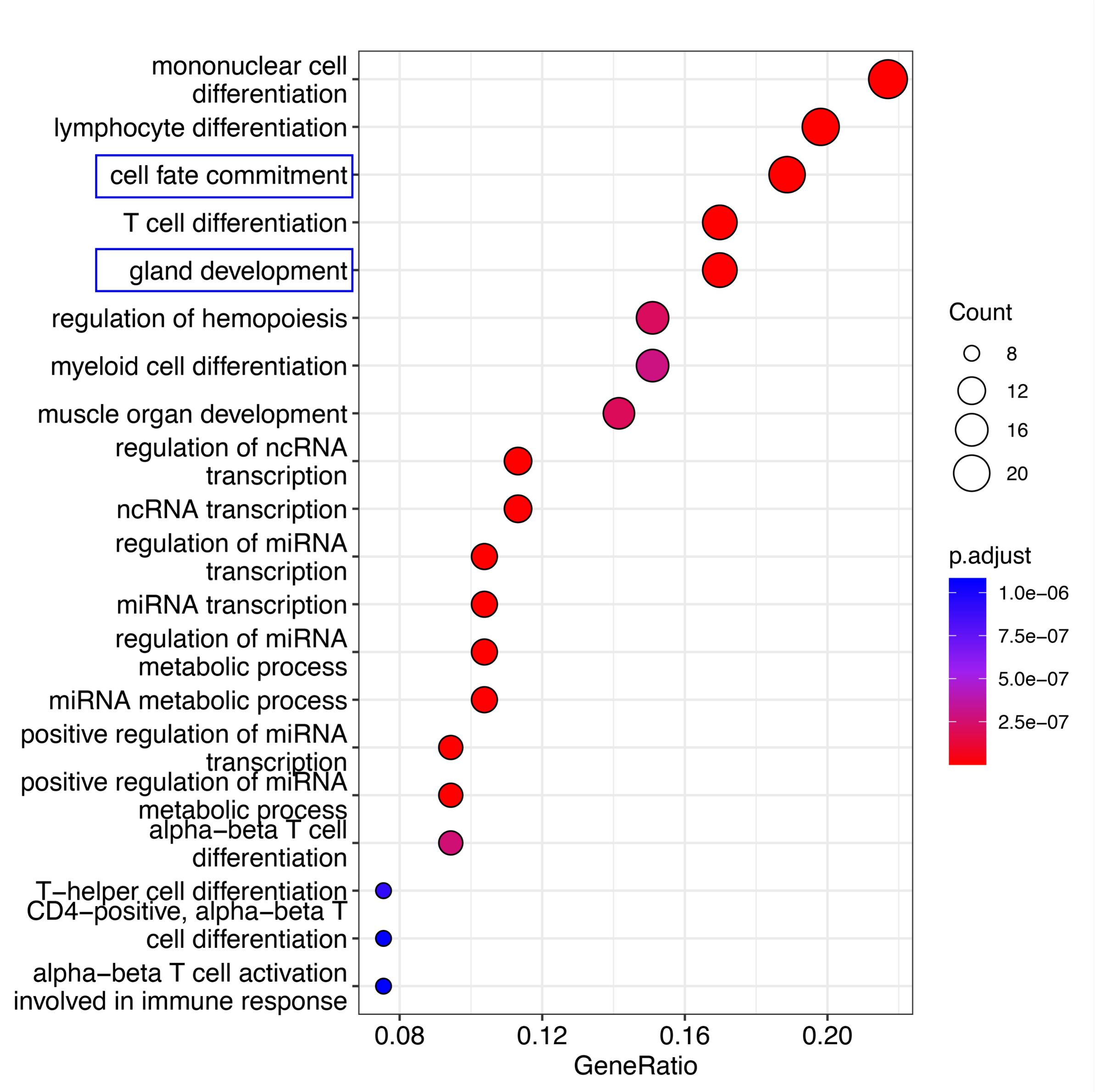

Figure S35. Enrichment analysis using ChIP-seq data from ICM cells and MII oocytes. We analyzed ChIP-seq data from [1], extracting significant peaks from ICM cells and MII oocytes (noncumulus) as the closest available proxy for polar bodies, mapping their sequences to motifs, and associating them with genes. Enrichment analysis revealed pathways like cell fate commitment and gland development, consistent with our original findings.

A.36 Supplementary Figure S36: Differential expression between ICM and cumulus cells.

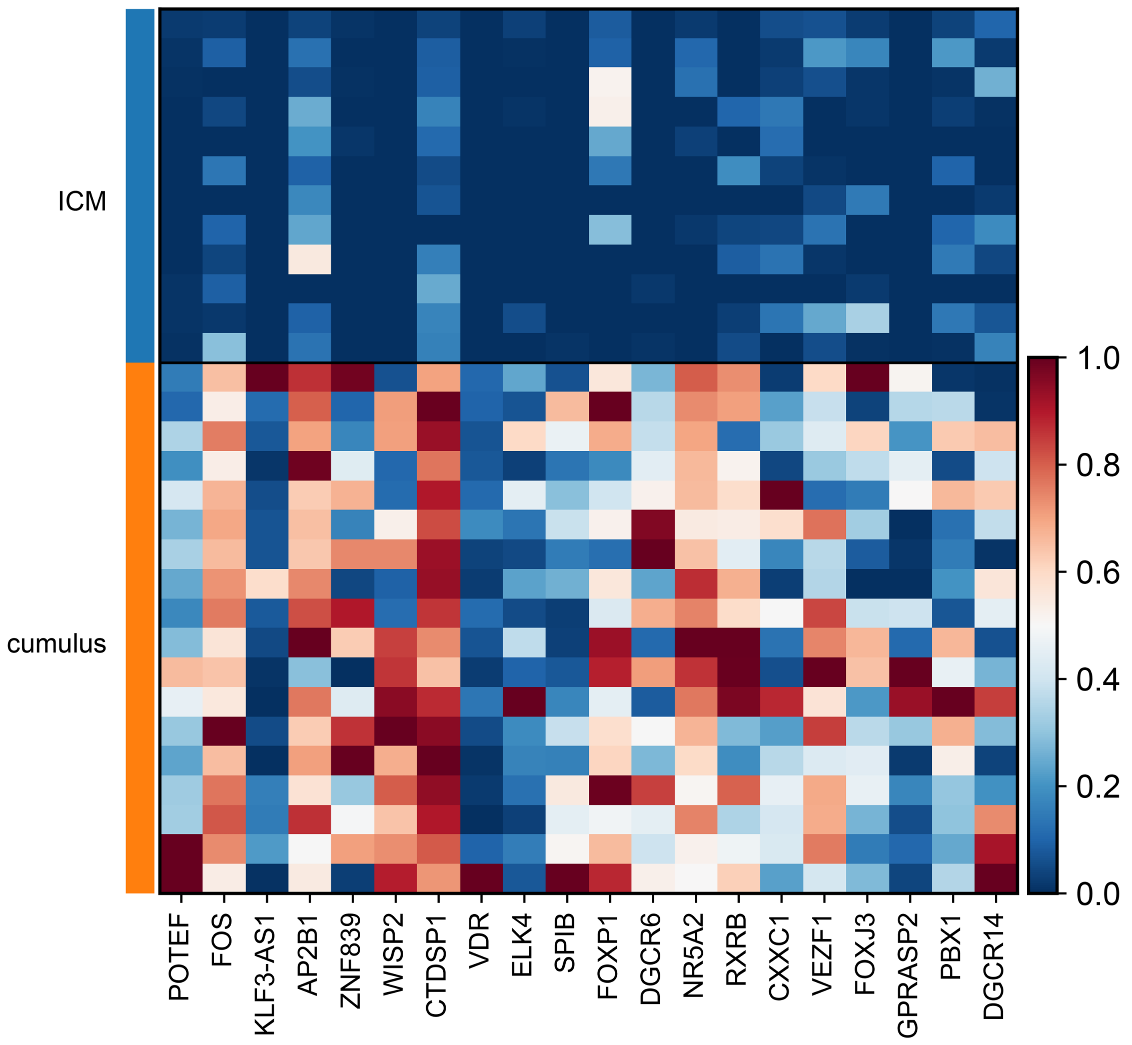

Figure S36. Differential expression between ICM and cumulus cells. We examined scRNA-seq data from ICM and cumulus cells [2, 3]. After preprocessing, we identified differentially expressed genes (DEGs) and compared them with motif-associated genes. Overlapping genes showed differential expression between ICM and cumulus cells, suggesting the biological relevance of the enriched motifs.

A.37 Supplementary Figure S37: Estimation of maternal contamination proportion.

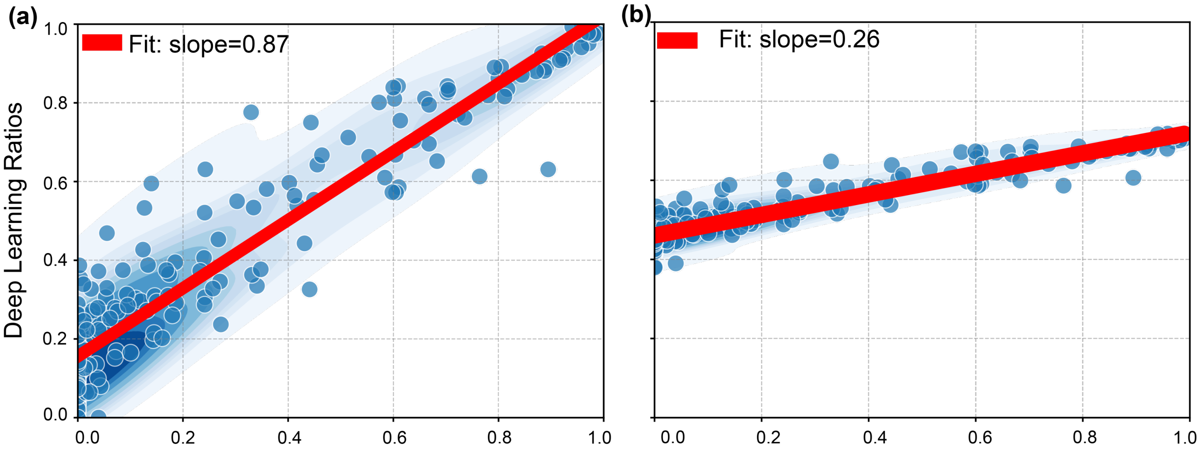

Figure S37. Estimation of maternal contamination proportion. We compared our MAP approach with a straightforward summarization method, such as calculating the proportion of reads with scores greater than 0.5 as an estimate of the contamination proportion. (a) MAP estimation. (b) summarization method.

B Supplementary Tables

B.1 Supplementary Table 1: DECNET's Architecture and Hyperparameters

Table S1. DECNET's architecture and hyperparameters.

Layer	Layer composition
conv1d	Conv1d $(5, 100, 10, \text{padding} = 4)$, ReLU, MaxPool1d $(2, \text{stride} = 2)$, Dropout (0.2)
self_attention	MultiheadAttention $(\text{embed\_dim} = 100, \text{num\_heads} = 4, \text{dropout} = 0.2)$, LayerNorm (100)
lstm	LSTM $(\text{input\_size} = 100, \text{hidden\_size} = 66)$
conv1d_2	Conv1d $(132, 100, 3, \text{padding} = 1)$, ReLU, MaxPool1d $(2, \text{stride} = 2)$, Dropout (0.2)
linear1	Linear $(1600, 512)$, ReLU, Dropout (0.2)
linear2	Linear $(512, 1)$, Sigmoid

B.2 Supplementary Table 2: Setting of Hyperparameters Used in the Training Process

Table S2. Setting of hyperparameters used in the training process.

Parameter	Value
Loss Function	BCELoss
Optimizer	SGD
Learning Rate	0.01
Momentum	0.9
Weight Decay	0
Nesterov	True
Learning Rate Scheduler	StepLR
Scheduler Step Size	5
Scheduler Gamma	0.5
Batch Size	128

B.3 Supplementary Table 3: Detailed Read Counts of Mixture Proportions Simulation

Table S3. Detailed read counts of mixture proportions simulation. See Methods.

Mixture Proportions (%)	S5 Reads Counts	S177 Reads Counts
0	1,000,000	0
10	900,000	100,000
20	800,000	200,000
30	700,000	300,000
40	600,000	400,000
50	500,000	500,000
60	400,000	600,000
70	300,000	700,000
80	200,000	800,000
90	100,000	900,000
100	0	1,000,000

B.4 Supplementary Table 4: Detailed Profiles of Filtered Moderate SECM Samples

Table S4. Detailed profiles of filtered moderate SECM samples (Overall 42 samples).

Samples	Original Counts	Filtered Counts (0.15)	Filtered Counts (0.2)	Contamination Level (%)
S19	5,902,528	281,356	525,774	57.20
S20	12,168,459	944,424	1,660,401	30.30
S25	3,827,085	257,678	458,038	30.90
S33	7,721,791	587,239	1,047,486	31.30
S37	9,988,097	918,054	1,607,694	16.40
S39	1,139,969	40,971	75,196	23.70
S41	6,078,437	505,329	900,490	13.60
S85	3,185,571	128,412	236,546	61.00
S89	7,314,223	517,429	913,416	37.70
S90	6,638,890	571,188	1,002,649	19.70
S93	2,695,133	178,076	308,271	29.50
S96	2,326,972	101,872	182,291	42.70
S99	5,377,871	470,104	817,220	11.40
S106	6,542,804	329,999	603,821	53.40
S124	2,724,440	140,810	247,716	37.20
S128	913,656	6,518	11,994	56.40
S134	2,102,564	88,816	161,195	39.50
S135	1,941,239	118,186	210,115	20.10
S138	1,987,841	65,671	117,599	37.50
S142	4,545,804	394,330	699,532	15.20
S150	3,278,963	127,901	237,862	58.10
S151	3,319,383	106,414	193,198	61.30
S155	5,882,254	517,861	897,847	18.70
S157	652,394	29,847	52,686	31.00
S161	4,372,441	351,585	609,026	26.10
S163	4,319,870	344,854	607,006	22.20
S164	3,467,061	203,719	354,190	15.20
S167	1,398,084	60,624	107,578	46.90
S168	5,893,497	406,865	710,750	27.20
S171	7,802,372	661,299	1,165,337	22.30
S174	6,247,627	452,900	805,399	32.80
S175	4,907,927	395,553	685,443	20.80
S176	4,818,527	388,926	675,155	27.20
S179	3,496,423	258,535	435,684	13.40
S180	5,131,279	268,437	481,800	53.30
S188	1,109,328	60,396	105,893	27.90
S191	1,932,896	52,165	94,612	59.50
S193	3,137,168	189,831	334,564	38.70
S199	1,145,212	58,226	98,959	21.50
S203	7,294,249	639,304	1,127,736	12.60
S207	6,480,228	447,421	786,123	33.90
S214	3,128,708	124,493	231,067	57.30

B.5 Supplementary Table 5: Detailed Profiles of Filtered Severe SECM Samples

Table S5. Detailed profiles of filtered severe SECM samples (Overall 40 samples).

Samples	Original Reads Counts	Filtered Reads Counts (Threshold 0.15)	Contamination Level (%)
S9	4,671,757	43,815	91.90
S10	1,708,891	8,183	69.60
S22	5,279,547	218,034	65.20
S28	5,027,178	199,412	64.30
S30	2,972,135	99,092	70.40
S34	2,742,162	25,485	99.80
S35	3,118,010	71,280	84.30
S48	657,527	2,568	84.30
S73	1,891,602	18,332	95.10
S75	1,409,930	11,603	92.60
S78	4,868,512	94,180	89.00
S81	4,926,389	146,119	77.70
S82	4,092,145	49,299	97.90
S83	3,863,356	44,662	97.60
S87	4,078,662	49,523	97.30
S94	1,128,432	5,429	93.80
S98	1,025,503	6,924	75.00
S101	1,800,846	37,442	71.20
S110	1,068,787	7,048	88.80
S115	1,489,409	21,688	83.30
S116	3,380,305	37,177	97.30
S118	808,099	5,118	81.10
S126	851,754	5,458	80.10
S132	772,595	3,819	66.80
S133	825,186	6,493	63.10
S146	1,790,489	23,562	86.30
S147	1,080,390	12,054	89.20
S154	6,081,279	252,740	66.70
S159	12,425,155	203,936	94.10
S177	8,072,316	99,357	99.40
S181	1,539,482	8,466	89.00
S183	1,552,341	23,694	87.10
S186	1,048,270	4,044	83.90
S198	1,189,436	22,187	66.30
S202	1,086,702	14,860	79.50
S208	819,889	4,656	75.50
S209	934,600	5,451	77.60
S210	20,467,013	409,593	90.80
S215	2,699,397	24,264	97.80
S219	1,193,614	17,137	76.20

B.6 Supplementary Table 6: Ratio of retained reads at zero contamination level using a threshold of 0.15.

Table S6. Ratio of retained reads at zero contamination level using a threshold of 0.15.

Sample	Counts	Retain Counts (0.15)	Contamination Level (%)	Retain Ratio (%)
PBAT_S24_B5	5,763,150	615,015	0.00	10.67
PBAT_S141_B12	9,831,333	1,110,396	0.00	11.29
PBAT_S184_B14	3,099,777	279,880	0.00	9.03
PBAT_S185_B14	8,710,767	1,076,657	0.00	12.36

B.7 Supplementary Table 7: Impact of DMR-Based Preselection on Read Retention in Samples with Low Contamination.

Table S7. Impact of DMR-Based Preselection on Read Retention in Samples with Low Contamination. This table quantifies the effect of applying DMR-based preselection on samples with contamination levels below 10%, showing the total reads, DMR reads, and retention ratio for each sample.

Sample	Total Reads	DMR Reads	Retain Ratio
PBAT_S4_B1	12,667,277	13,668	0.10790006
PBAT_S5_B1	28,300,638	29,375	0.10380366
PBAT_S7_B1	9,509,273	8,201	0.08624213
PBAT_S24_B5	5,763,150	5,690	0.09873073
PBAT_S36_B5	5,389,769	5,135	0.0952731
PBAT_S40_B5	5,249,129	3,807	0.07252632
PBAT_S45_B5	8,626,404	6,721	0.07791186
PBAT_S49_B5	5,235,634	5,765	0.11011651
PBAT_S50_B5	6,634,379	6,292	0.09483932
PBAT_S51_B5	4,432,428	4,091	0.09229704
PBAT_S53_B5	21,530,819	19,575	0.09091619
PBAT_S91_B10	5,829,319	7,623	0.13076999
PBAT_S92_B10	2,962,464	3,166	0.10687005
PBAT_S102_B10	8,321,588	8,850	0.10634989
PBAT_S107_B11	15,504,019	17,650	0.11381445
PBAT_S122_B11	5,978,001	6,791	0.11359985
PBAT_S123_B11	11,778,022	13,729	0.11656456
PBAT_S125_B11	6,971,355	7,721	0.11075232
PBAT_S126_B11	5,846,303	6,546	0.11193687
PBAT_S127_B11	12,740,294	14,107	0.11072743
PBAT_S131_B12	7,149,659	8,932	0.12492904
PBAT_S136_B12	2,628,347	3,238	0.12311953
PBAT_S141_B12	9,831,333	13,191	0.13417306
PBAT_S144_B12	789,857	11,922	0.150993152
PBAT_S145_B12	13,214,770	15,063	0.11398609
PBAT_S149_B12	2,261,229	2,750	0.12161528
PBAT_S166_B13	5,874,226	6,328	0.10772483
PBAT_S170_B13	13,363,515	14,180	0.10610981
PBAT_S184_B14	3,099,777	3,401	0.10971757
PBAT_S185_B14	8,710,676	9,763	0.11207968
PBAT_S187_B14	8,026,286	10,850	0.13518083
PBAT_S190_B14	7,538,763	9,134	0.12116046
PBAT_S197_B14	5,687,967	7,310	0.12851692
PBAT_S200_B15	5,666,044	5,566	0.09823432
PBAT_S201_B15	4,170,117	3,929	0.09421788
PBAT_S205_B15	2,051,844	2,153	0.104938
PBAT_S211_B15	10,108,085	10,942	0.10824998
PBAT_S218_B16	14,867,608	13,252	0.08913377

References

[1] Weikun Xia et al. "Resetting histone modifications during human parental-to-zygotic transition". In: *Science* 365.6451 (2019), pp. 353–360.

[2] Liying Yan et al. "Single-cell RNA-Seq profiling of human preimplantation embryos and embryonic stem cells". In: *Nature structural & molecular biology* 20.9 (2013), pp. 1131–1139.

[3] Yaoyao Zhang et al. “Transcriptome landscape of human folliculogenesis reveals oocyte and granulosa cell interactions”. In: *Molecular cell* 72.6 (2018), pp. 1021–1034.
